# Supplementary material for: Proteasome inhibition enhances lysosome-mediated targeted protein degradation
Source: Cell Death Dis. 2026 May 11;17(1):614. doi: 10.1038/s41419-026-08835-6 (PMC13328728; doi:10.1038/s41419-026-08835-6)
Supplement: Supplementary file 1 — Supplementary Figures [file 41419_2026_8835_MOESM1_ESM.pptx]

## Slide 1
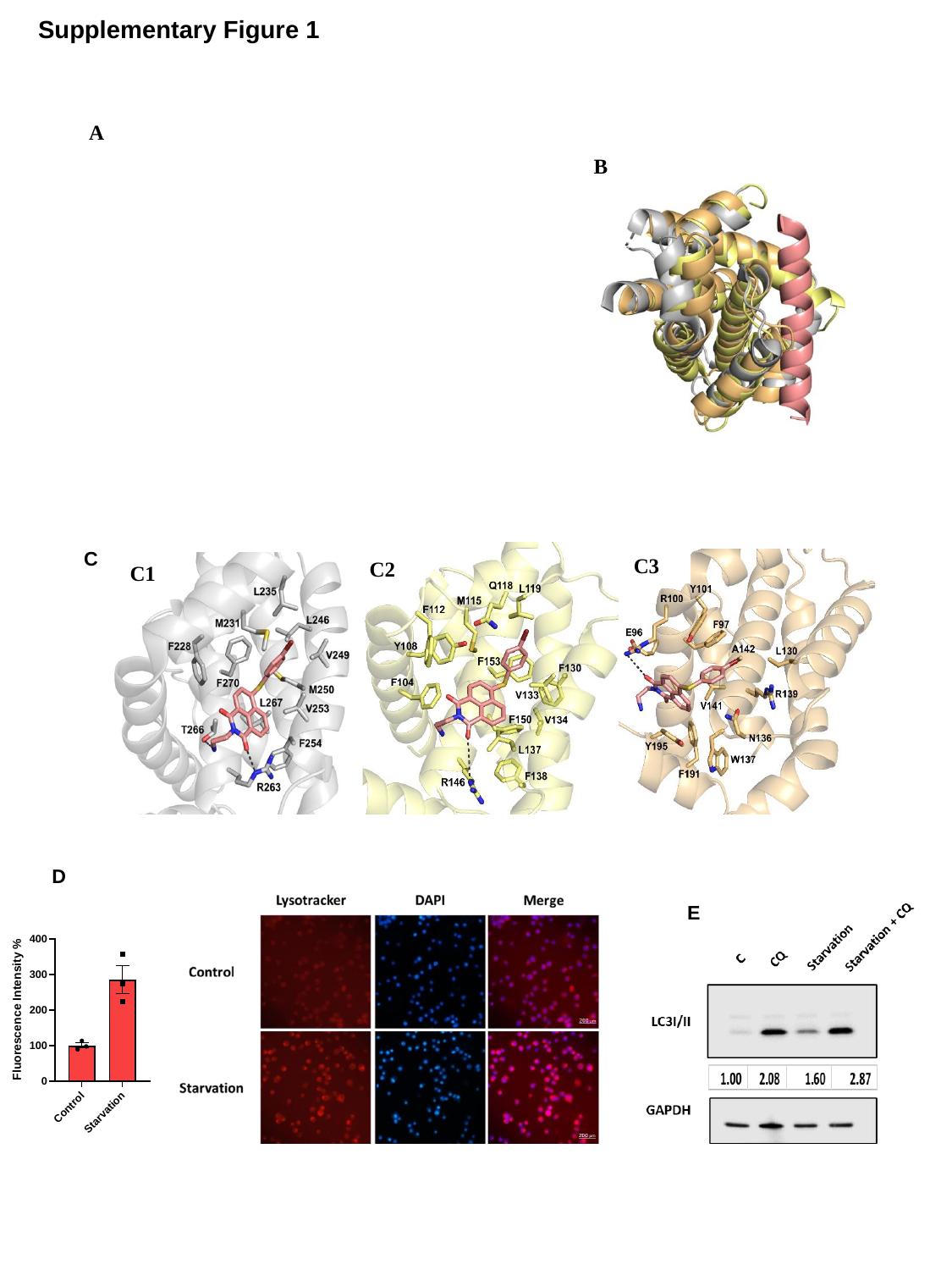

Supplementary Figure 1
A
B
C
C2
C1
C3
D
E

## Slide 2
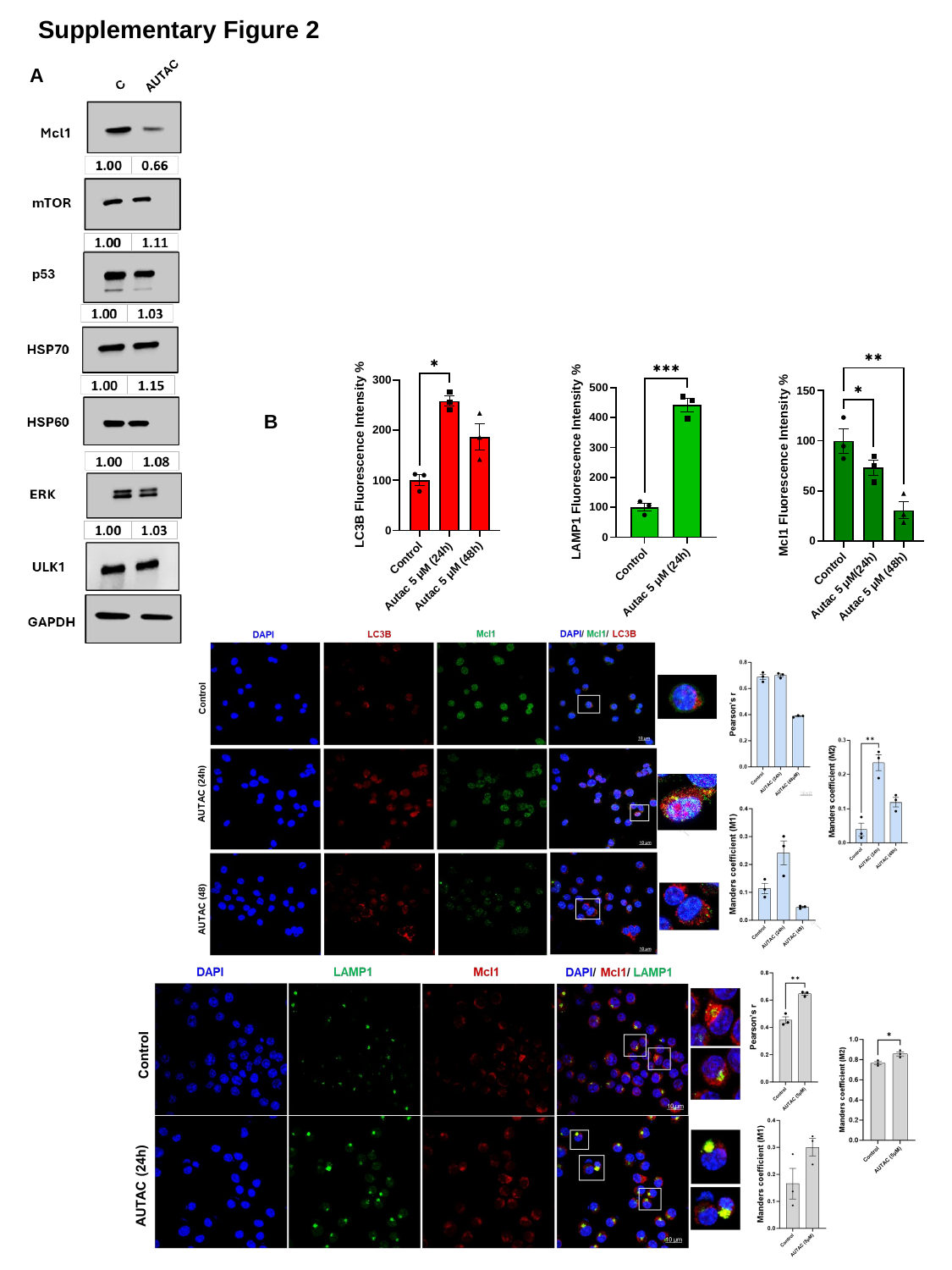

Supplementary Figure 2
A
B

## Slide 3
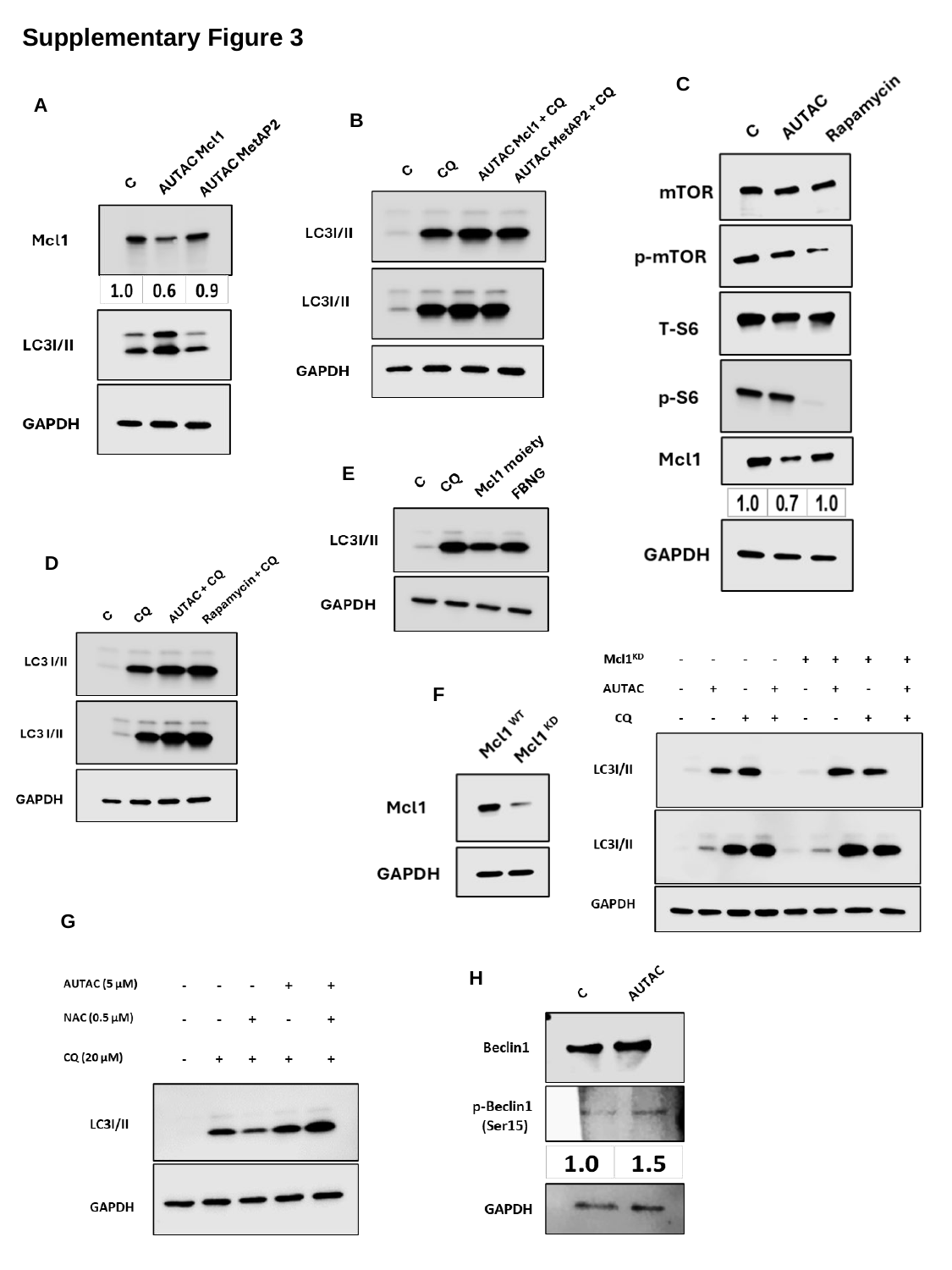

Supplementary Figure 3
C
A
B
E
D
F
G
H

## Slide 4
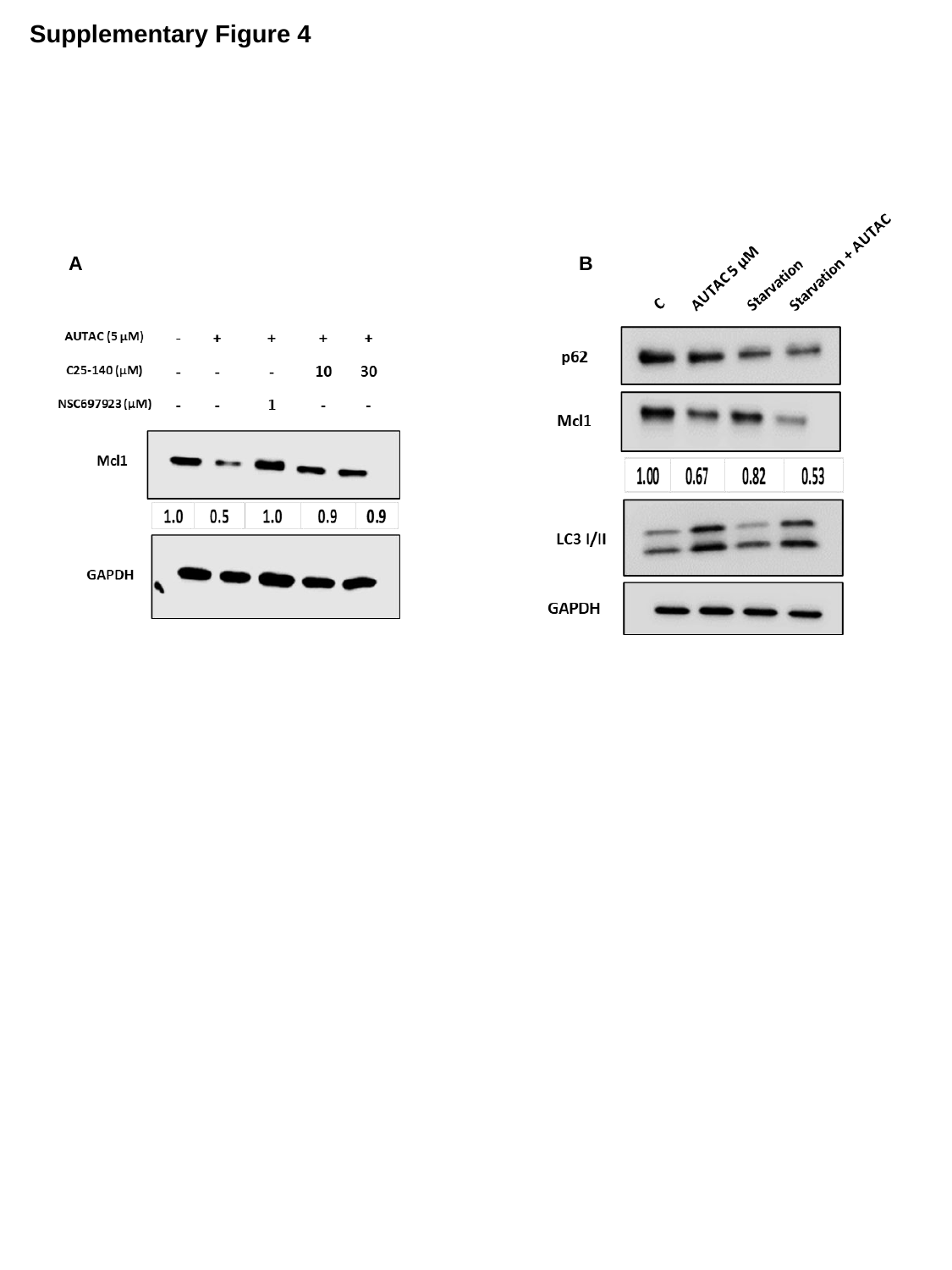

Supplementary Figure 4
B
A

## Slide 5
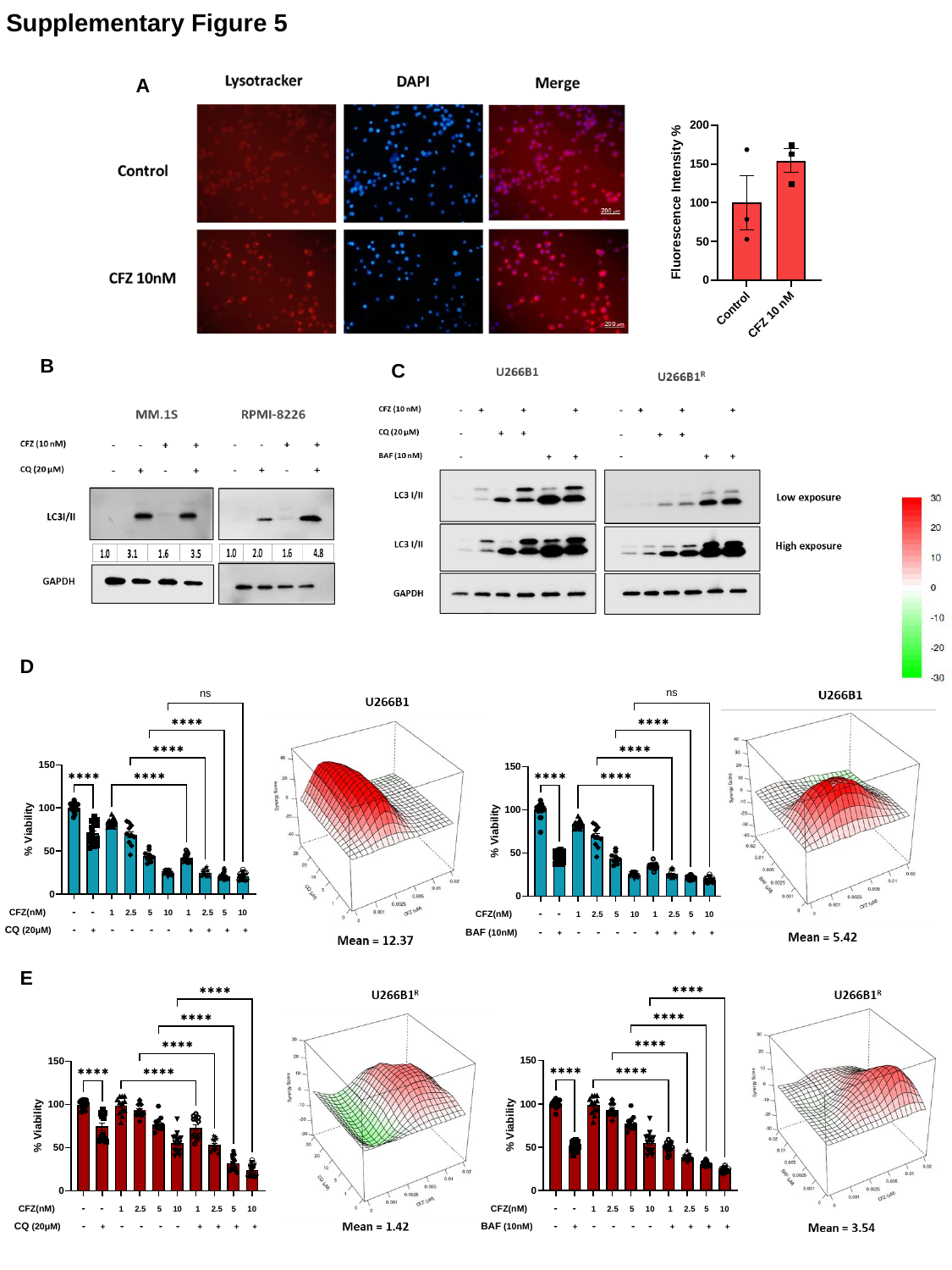

Supplementary Figure 5
A
B
C
D
E

## Slide 6
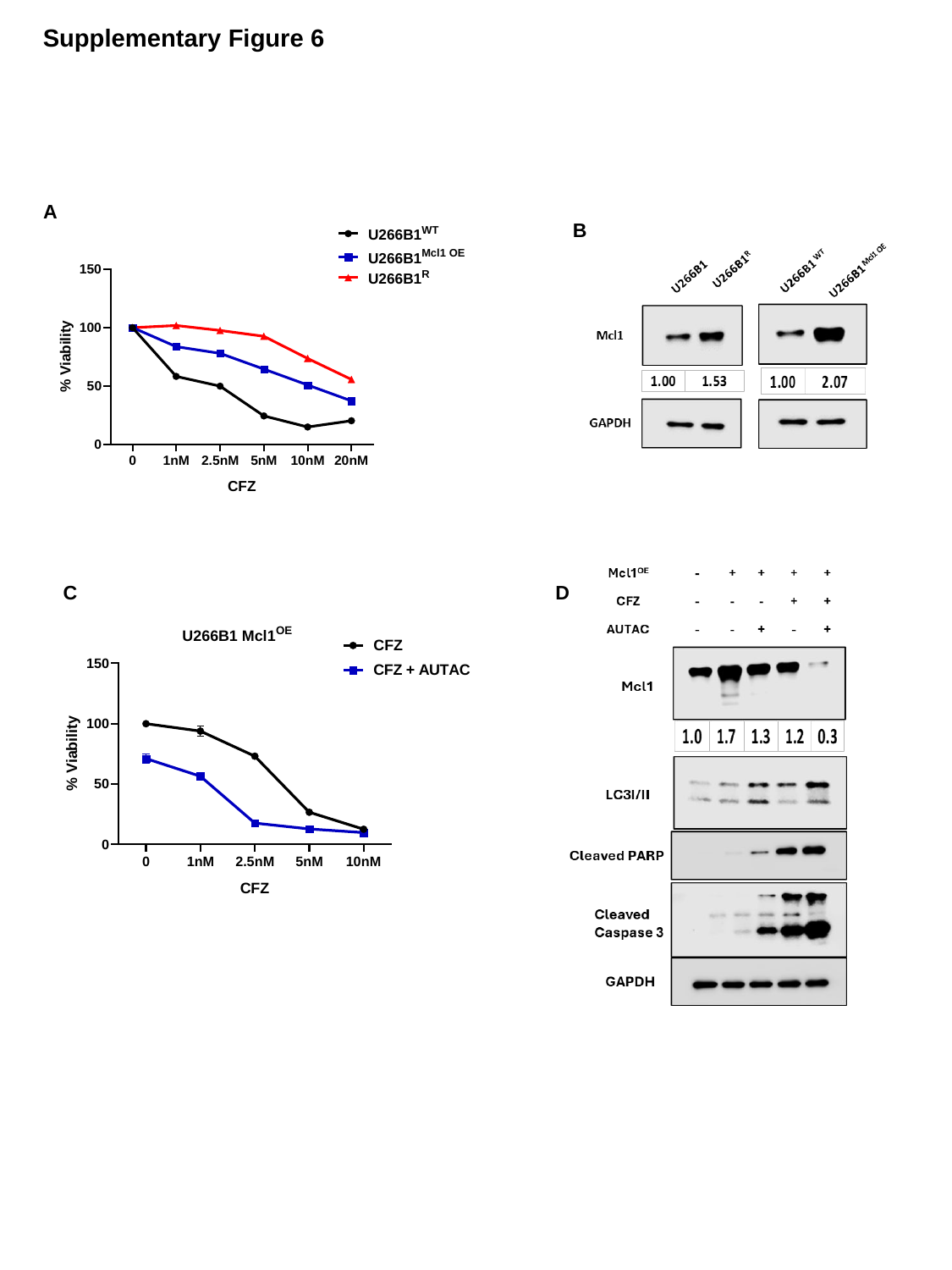

Supplementary Figure 6
A
B
D
C
