# Supplementary material for: Proteasome inhibition enhances lysosome-mediated targeted protein degradation
Source: Cell Death Dis. 2026 May 11;17(1):614. doi: 10.1038/s41419-026-08835-6 (PMC13328728; doi:10.1038/s41419-026-08835-6)
Supplement: Supplementary file 2 — Supplementary Figure Legends [file 41419_2026_8835_MOESM2_ESM.pdf]

Supplementary Figure 1. Structural comparison and docking of AUTAC warhead in Mcl1, Bcl2, and Bclxl. (A) Sequence alignment of human Mcl1, Bcl2 and Bclxl; **(B)** Structural overlay of Mcl1 vs Bcl2 vs Bclxl BH3- binding grooves. Mcl1 (PDB ID: 2PQK), Bcl2 (PDB ID: 2XA0), Bclxl (PDB ID 1R2D) and BH3-peptides are shown as grey, yellow, orange and salmon cartoons respectively. **(C)** Highest-ranked docking pose of the warhead in (**C1**) Mcl1, (**C2**) Bcl2, and (**C3**) Bclxl. Proteins are shown as cartoons (Mcl1 grey, Bcl2 yellow, Bclxl orange). The warhead is shown as salmon sticks. Key pocket residues are shown as sticks, and hydrogen-bonding interactions are indicated by dashed lines. **(D)** Representative florescence microscopy images and their quantification for U266B1 cells subjected to starvation for 24h, then the cells stained with DAPI and lysotracker stain. All images were generated under the same magnification (20X). **(E)** Western blot analysis for LC3I/II protein in U266B1 starved and treated with CQ (20 μM) for 24h. GAPDH was used as the loading control.

Three biological replicates for each cell line were used to perform Western blotting. The uncropped blots are shown in the Supplemental Materials.

Supplementary Figure 2. AUTAC promotes lysosomal and autophagic association of Mcl1 in U266B1 cells. (A) Western blot analysis for mTOR, p53, HSP70, HSP60, ERK, and ULK1 proteins in U266B1 treated with AUTAC 5 μM for 24h. GAPDH was used as the loading control. **(B)** Confocal images with their quantification for Mcl1, LAMP1 and LC3B proteins in U266B1 cells treated with AUTAC (5 μM) for 24 and 48h (scale 10 μM).

Three biological replicates for each cell line were used to perform Western blotting. The uncropped blots are shown in the Supplemental Materials. Statistical significance of each condition compared to the indicated control or treatment was determined using unpaired Student's t-test or two-way ANOVA with Tukey or Sidak post hoc tests, as appropriate. Data are represented as mean ± SEM. Significance levels are indicated as follows: * $p < 0.05$, ** $p < 0.01$, *** $p < 0.001$ and **** $p < 0.0001$.

Supplementary Figure 3. AUTAC selectively degrades Mcl1 and subsequently induces autophagy through a Beclin1–dependent axis.(A) Western blot analysis for Mcl1, and LC3I/II proteins in U266B1 treated with Mcl1 AUTAC (5μM) or MetAP2 AUTAC (10μM) for 24h. GAPDH was used as the loading control. **(B)** Western blot analysis for LC3I/II protein in U266B1 treated with CQ (20 μM) and/or Mcl1 AUTAC (5μM) or MetAP2 AUTAC (10μM) for 24h. GAPDH was used as the loading control. **(C)** Western blot analysis for Mcl1, mTOR, p-mTOR, T-S6, and p-S6 proteins in U266B1 treated with Mcl1 AUTAC (5μM) and rapamycin (100nM) for 24h. GAPDH was used as the loading control. **(D)** Western blot analysis for LC3I/II protein in U266B1 treated with CQ (20 μM) and/or Mcl1 AUTAC (5μM), or rapamycin (100nM) for 24h. GAPDH was used as the loading control. **(E)** Western blot analysis for LC3I/II protein in U266B1 treated with CQ (20 μM) and/or Mcl1 moiety (10μM), or FBNG (10μM) for 24h. GAPDH was used as the loading control. **(F)** Western blot analysis for Mcl1, and LC3I/II proteins in U266B1^{WT}, and U266B1 Mcl1 ^{KD} treated with CQ (20 μM) and/or AUTAC (5μM) for 24h. GAPDH was used as the loading control. **(G)** Western blot analysis for LC3I/II protein in U266B1 treated with CQ

(20 μM), AUTAC (5 μM), and N-acetyl cysteine (NAC) (0.5 μM) for 24h. GAPDH was used as the loading control. **(H)** Western blot analysis for Beclin1, and p-Beclin1 (Ser15) proteins in U266B1 treated with AUTAC (5 μM) for 24h. GAPDH was used as the loading control.

Three biological replicates for each cell line were used to perform Western blotting. The uncropped blots are shown in the Supplemental Materials.

Supplementary Figure 4. Autophagy induction via starvation augments the Mcl1 degradation ability of AUTAC. (A) Western blot analysis for Mcl1 protein in U266B1 treated with AUTAC (5 μM), NSC697923 (1μM) and/or C25-140 (10 and 30 μM) for 24h. GAPDH was used as the loading control. **(B)** Western blot analysis for p62, Mcl1, and LC3I/II proteins in U266B1 starved and treated with AUTAC (5 μM) for 24h. GAPDH was used as the loading control.

Three biological replicates for each cell line were used to perform Western blotting. The uncropped blots are shown in the Supplemental Materials.

Supplementary Figure 5. Carfilzomib induces a cytoprotective autophagy in multiple myeloma cells. (A) Representative florescence microscopy images with their quantification for U266B1 cells treated with carfilzomib (10 nM) for 24h, then the cells stained with DAPI and lysotracker stain. All images were generated under the same magnification (20X). **(B)** Western blot analysis for LC3I/II protein in MM.1S and RPMI-8226 cells treated with carfilzomib (10 nM) and/or CQ (20 μM) for 16h. GAPDH was used as the loading control. **(C)** Western blot analysis for LC3I/II protein in U266B1 and U266B1^{R} treated with carfilzomib (10 nM) and/or CQ (20 μM) or bafilomycin (10 nM) for 24h. GAPDH was used as the loading control. **(D and E)** The percentage of cellular viability for **(D)** U266B1 and **(E)** U266B1^{R} cells treated with CQ (20 μM) or bafilomycin (10 nM) for 72h, then the indicated concentrations of CFZ were added for the last 24h. The synergy between CQ, or BAF and CFZ were determined using Bliss score.

Three biological replicates for each cell line were used to perform cell viability assay, and Western blotting. In each independent replicate of the synergy assay, a single well was used for each drug combination. The uncropped blots are shown in the Supplemental Materials. ns indicates non-statistical significance, while **** $p < 0.0001$ indicates statistical significance of each condition compared to the indicated control/treatment as determined using two-way ANOVA with Sidak's post hoc test.

Supplementary Figure 6. Mcl1 contributes to proteasome inhibitor resistance in multiple myeloma cells. (A) Viability of U266B1^{WT}, U266B1^{R}, and U266B1 Mcl1 overexpression (OE) cells treated with the indicated concentrations of CFZ for 24h. **(B)** Western blot analysis for Mcl1 protein in U266B1^{WT}, U266B1^{R}, and U266B1 Mcl1 ^{OE}. GAPDH was used as the loading control. **(C)** The percentage of cellular viability for U266B1^{WT} and U266B1 Mcl1^{OE} cells treated with AUTAC (5 μM) for 72h, then the indicated concentrations of CFZ were added for the last 24h. **(D)** Western blot analysis for Mcl1, LC3I/II, cleaved PARP, and cleaved caspase 3 proteins in

U266B1^{WT} and U266B1 Mcl1^{OE} cells treated with AUTAC (5 μM) for 48h, then CFZ (10 nM) was added for the last 24h. GAPDH was used as the loading control.

Three biological replicates for each cell line were used to perform cell viability assay, and Western blotting. The uncropped blots are shown in the Supplemental Materials.
