# Supplementary material for: Proteasome inhibition enhances lysosome-mediated targeted protein degradation
Source: Cell Death Dis. 2026 May 11;17(1):614. doi: 10.1038/s41419-026-08835-6 (PMC13328728; doi:10.1038/s41419-026-08835-6)
Supplement: Supplementary file 3 — Uncropped Blots [file 41419_2026_8835_MOESM3_ESM.pptx]

## Slide 1
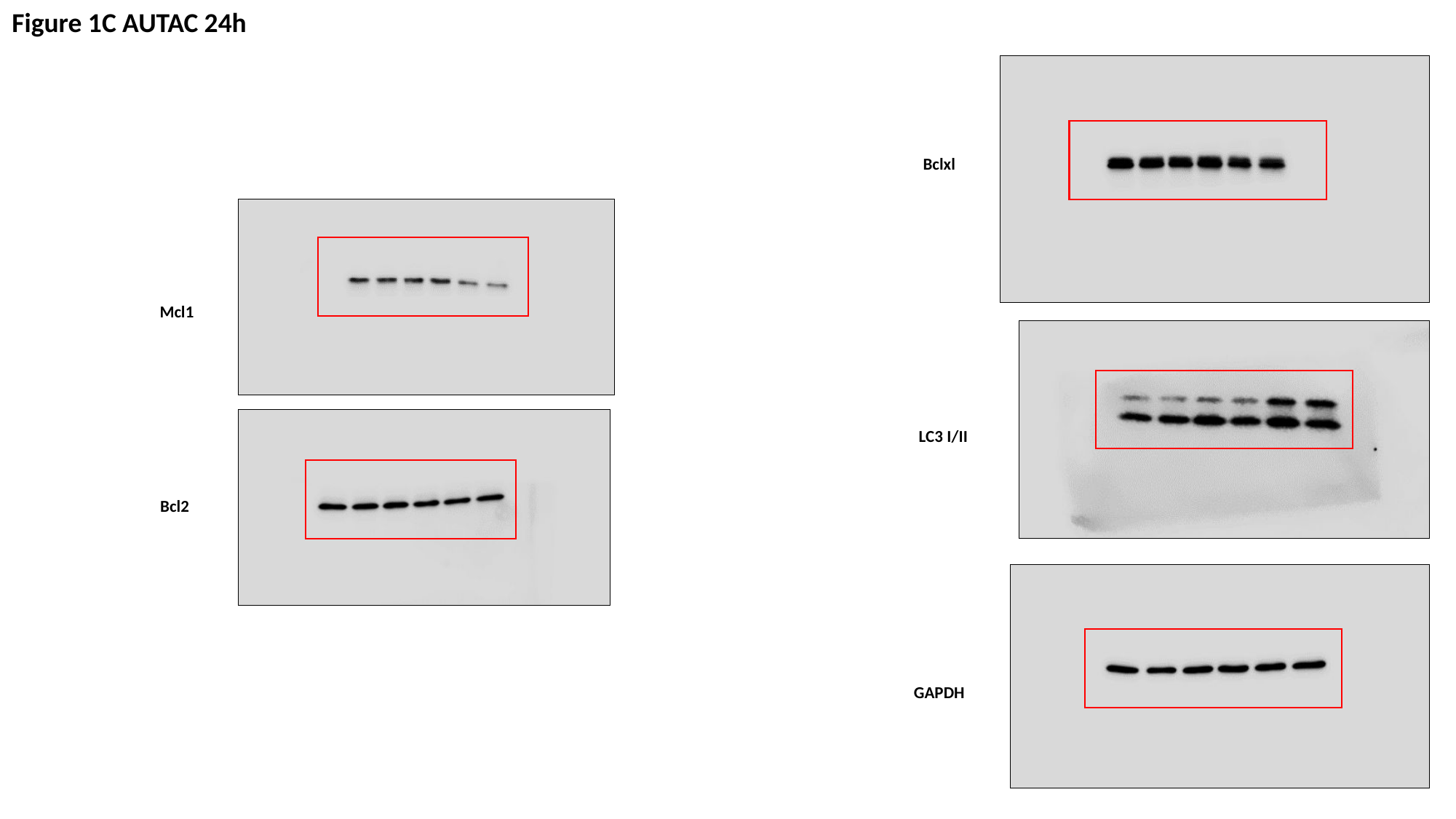

Figure 1C AUTAC 24h
Bclxl
Mcl1
LC3 I/II
Bcl2
GAPDH

## Slide 2
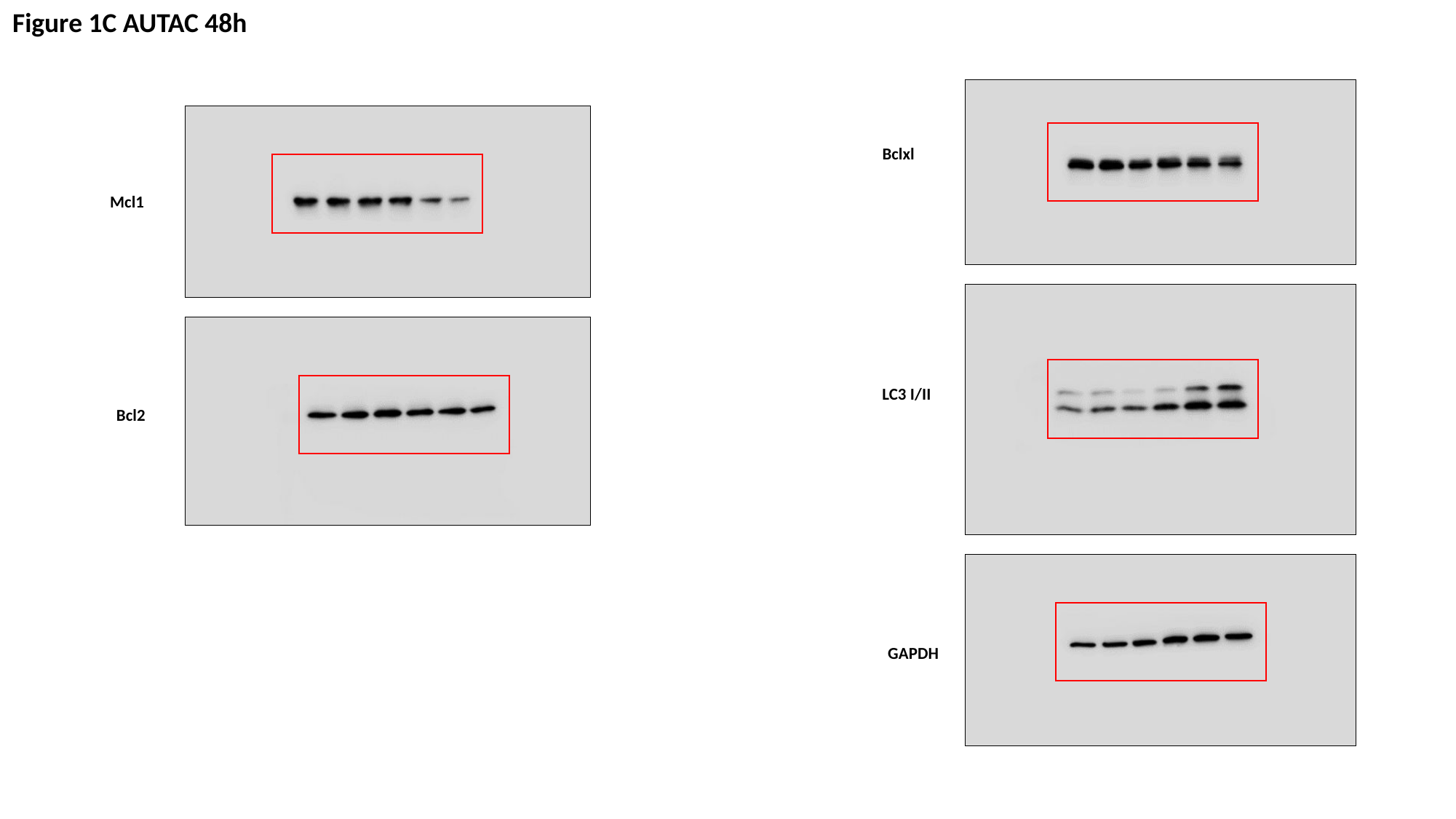

Figure 1C AUTAC 48h
Bclxl
Mcl1
LC3 I/II
Bcl2
GAPDH

## Slide 3
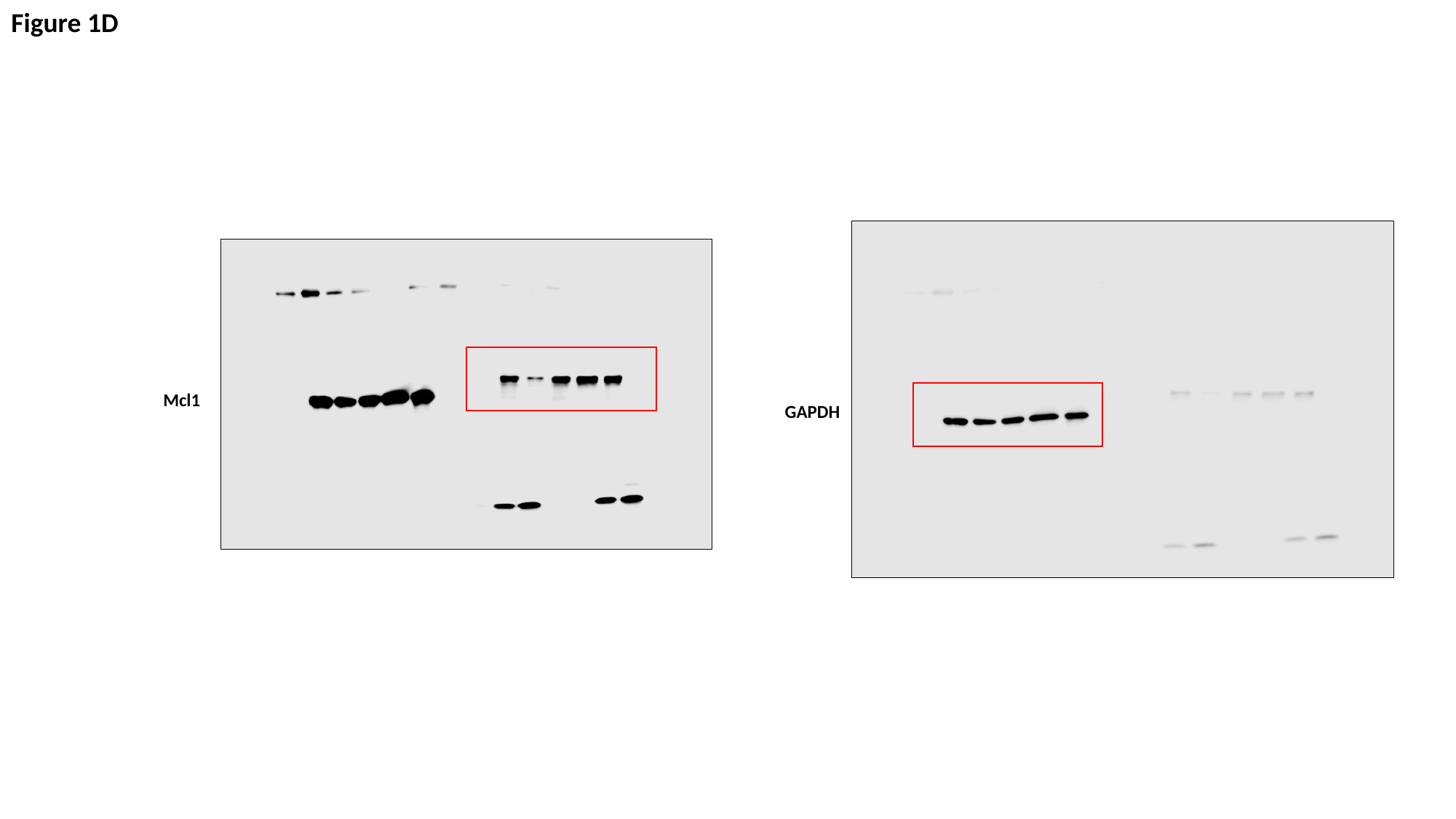

Figure 1D
Mcl1
GAPDH

## Slide 4
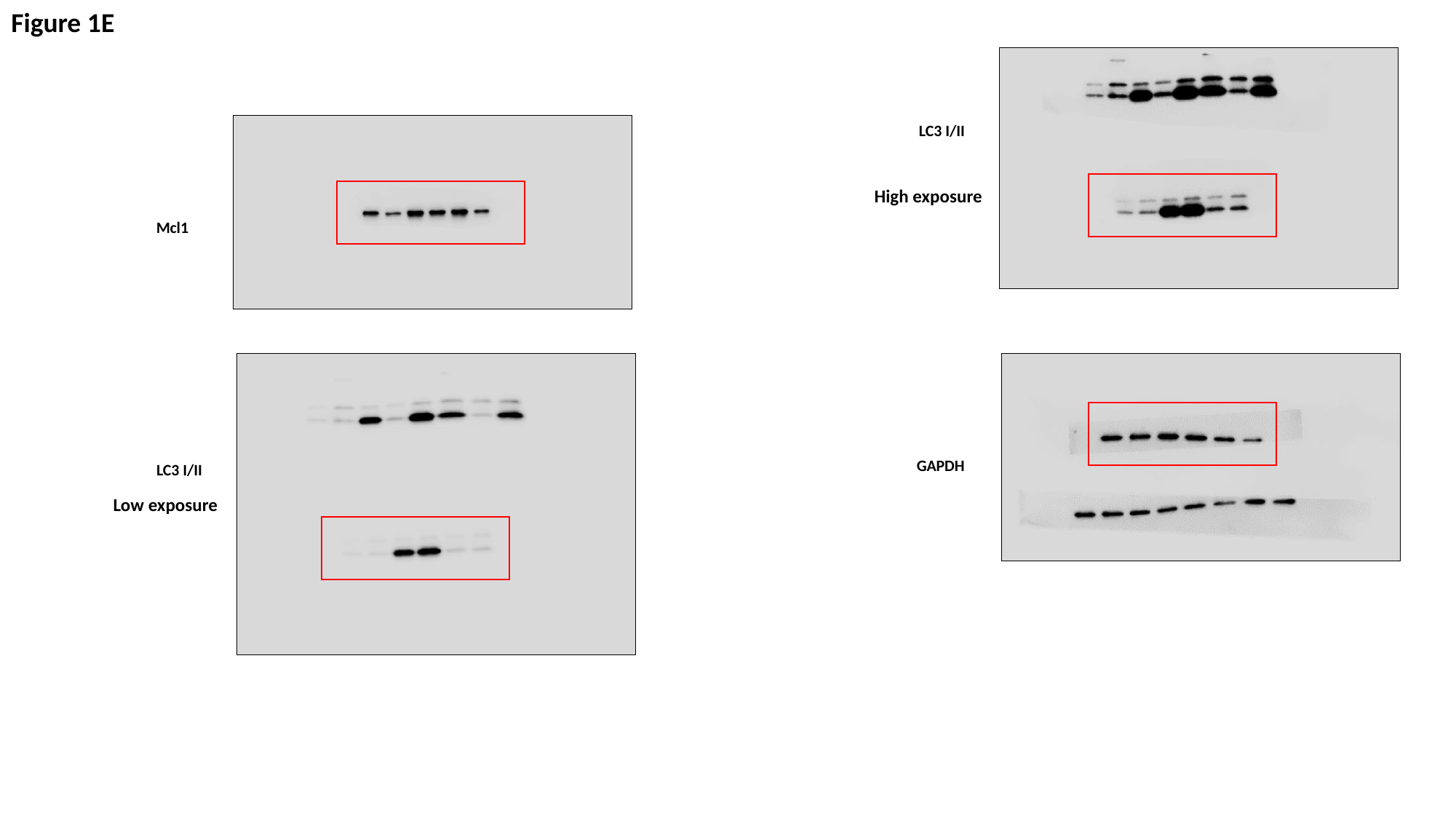

Figure 1E
LC3 I/II
High exposure
Mcl1
GAPDH
LC3 I/II
Low exposure

## Slide 5
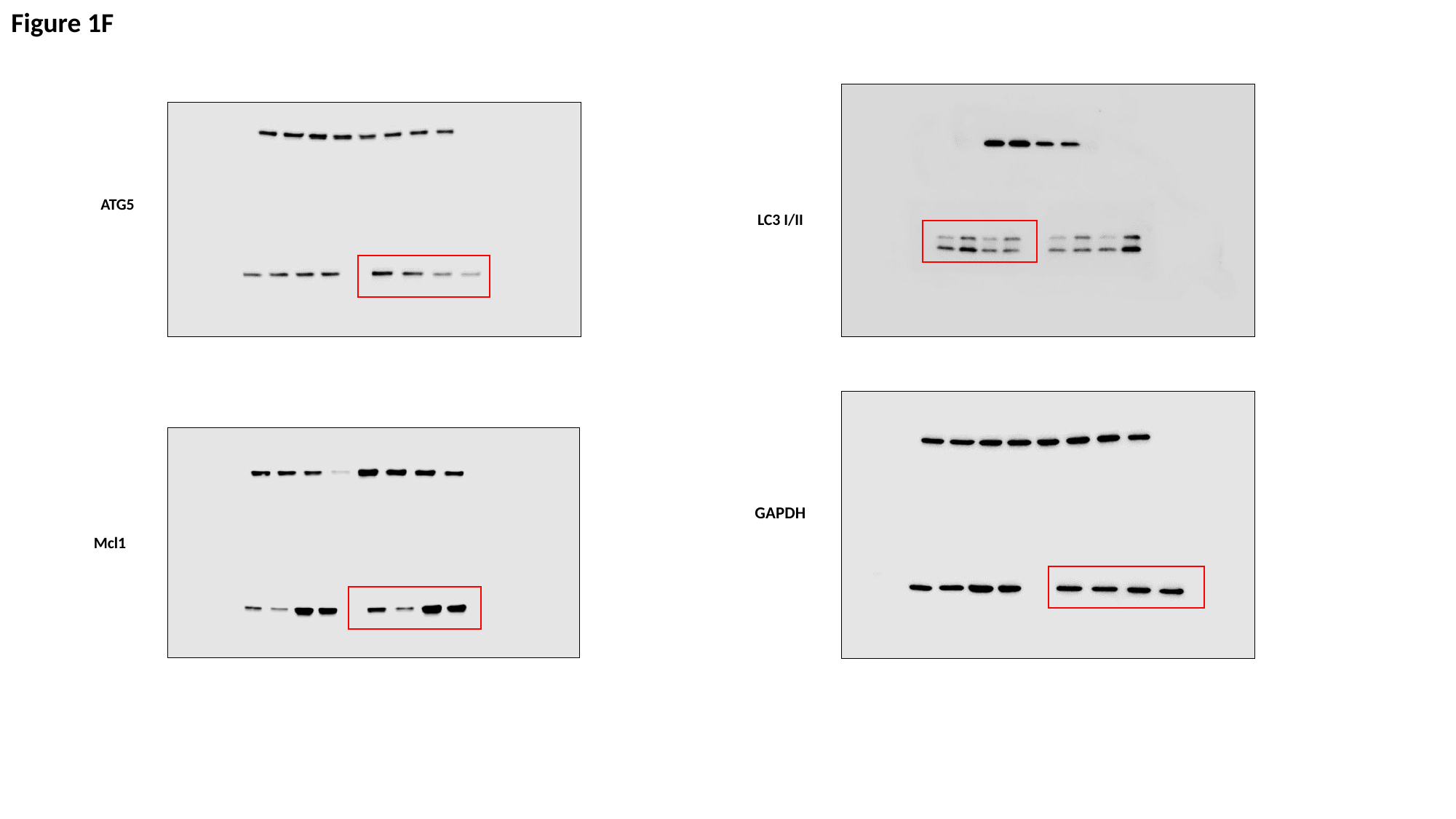

Figure 1F
ATG5
LC3 I/II
GAPDH
Mcl1

## Slide 6
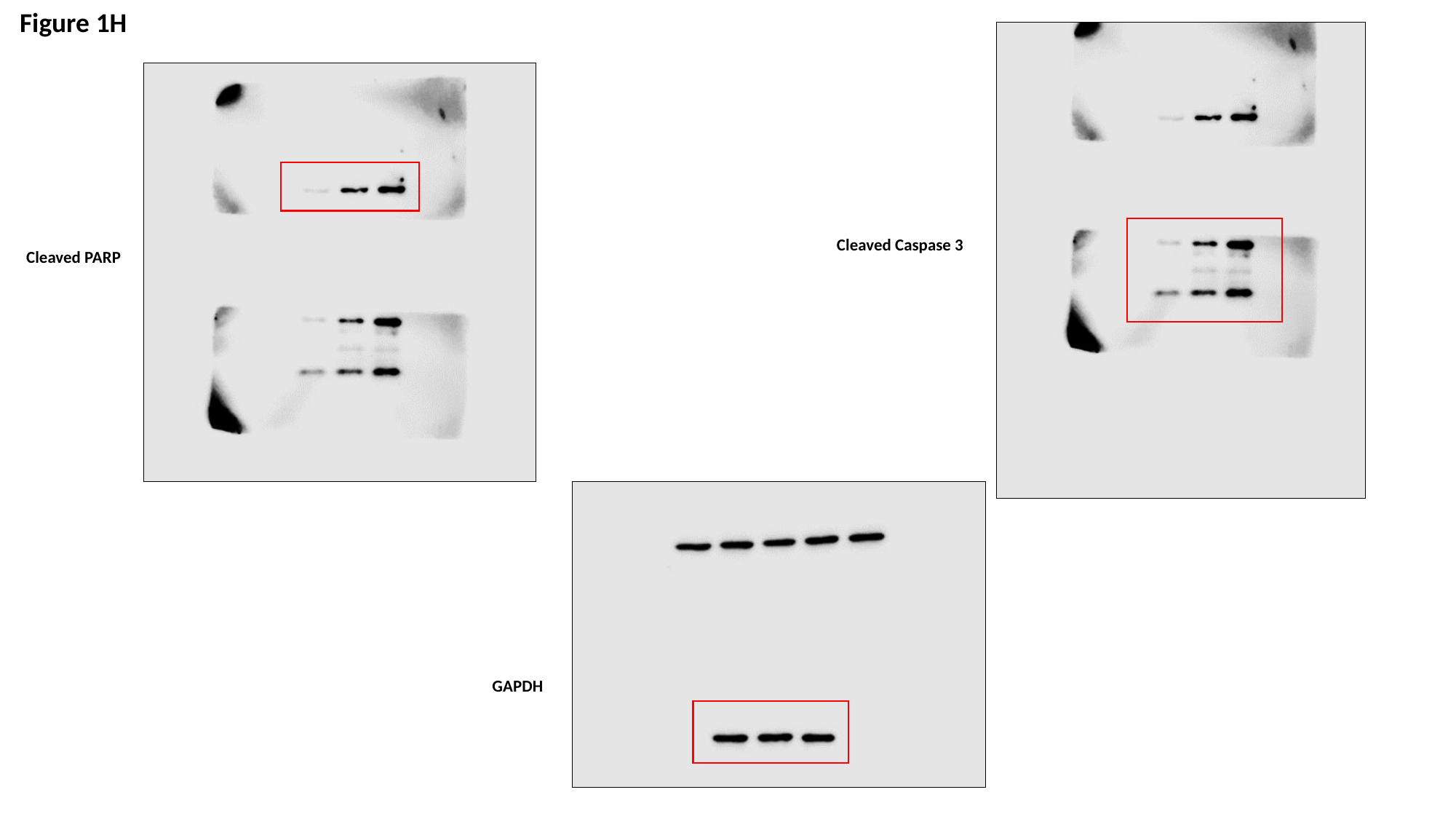

Figure 1H
Cleaved Caspase 3
Cleaved PARP
GAPDH

## Slide 7
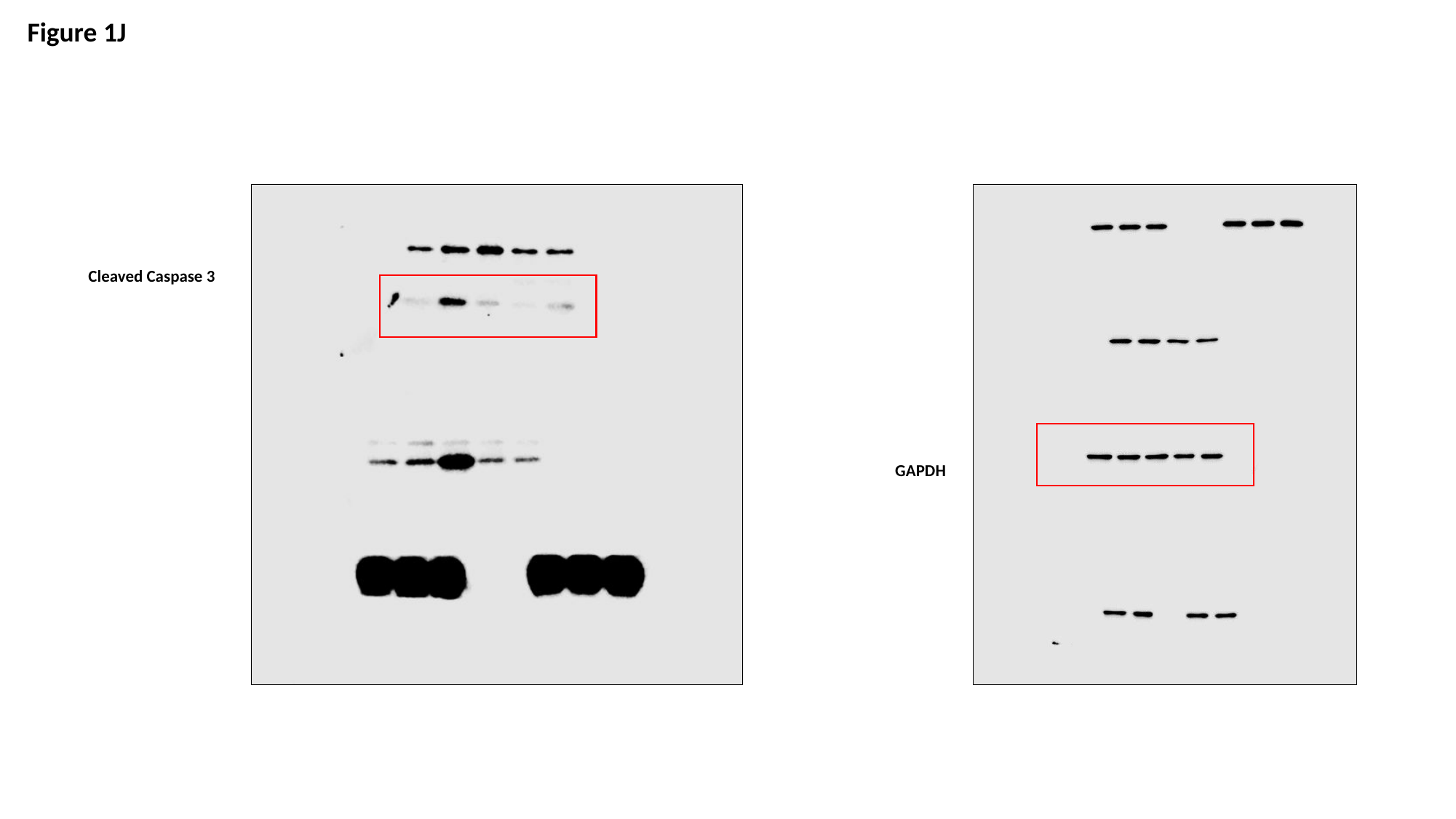

Figure 1J
Cleaved Caspase 3
GAPDH

## Slide 8
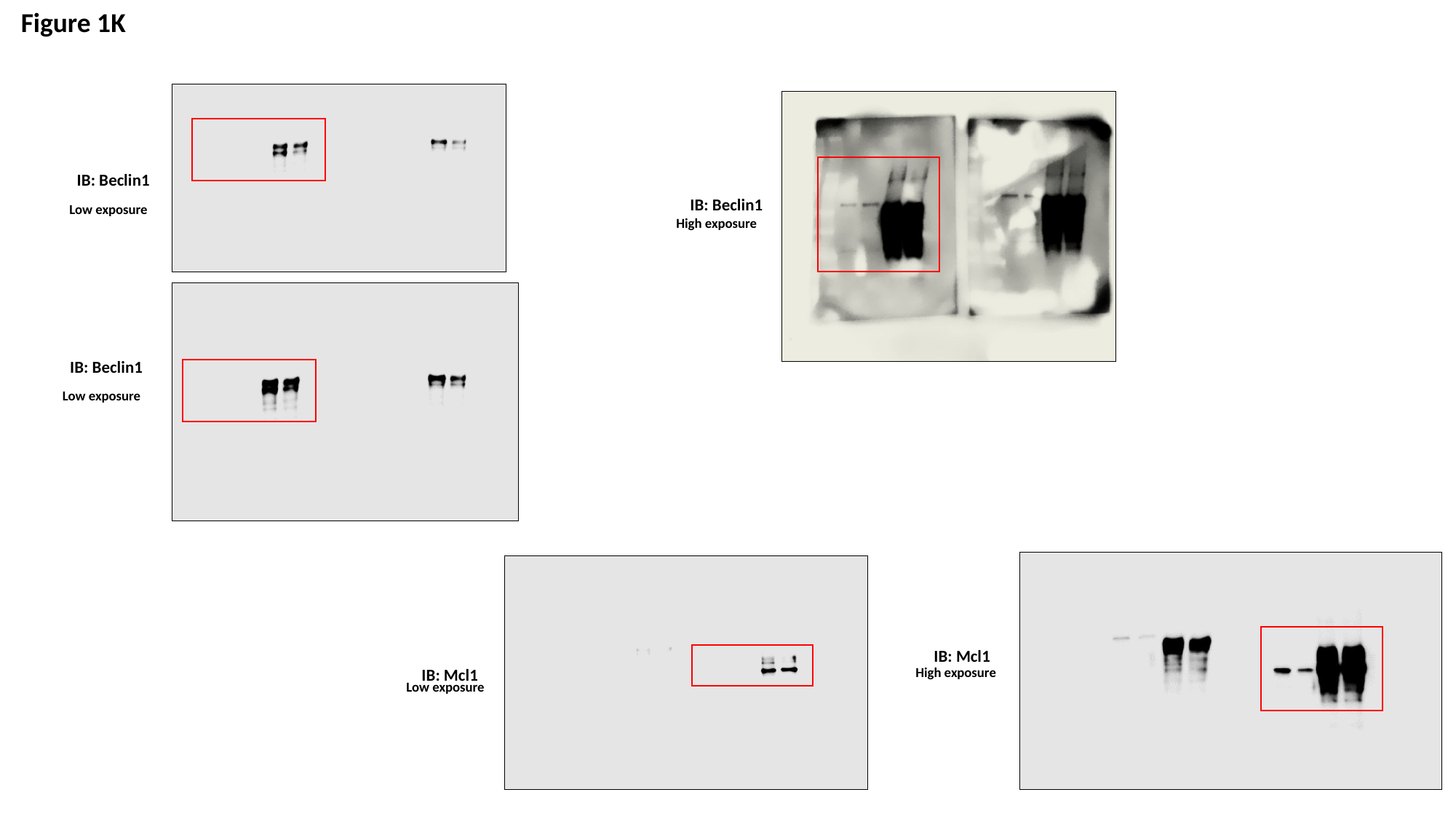

Figure 1K
IB: Beclin1
IB: Beclin1
Low exposure
High exposure
IB: Beclin1
Low exposure
IB: Mcl1
IB: Mcl1
High exposure
Low exposure

## Slide 9
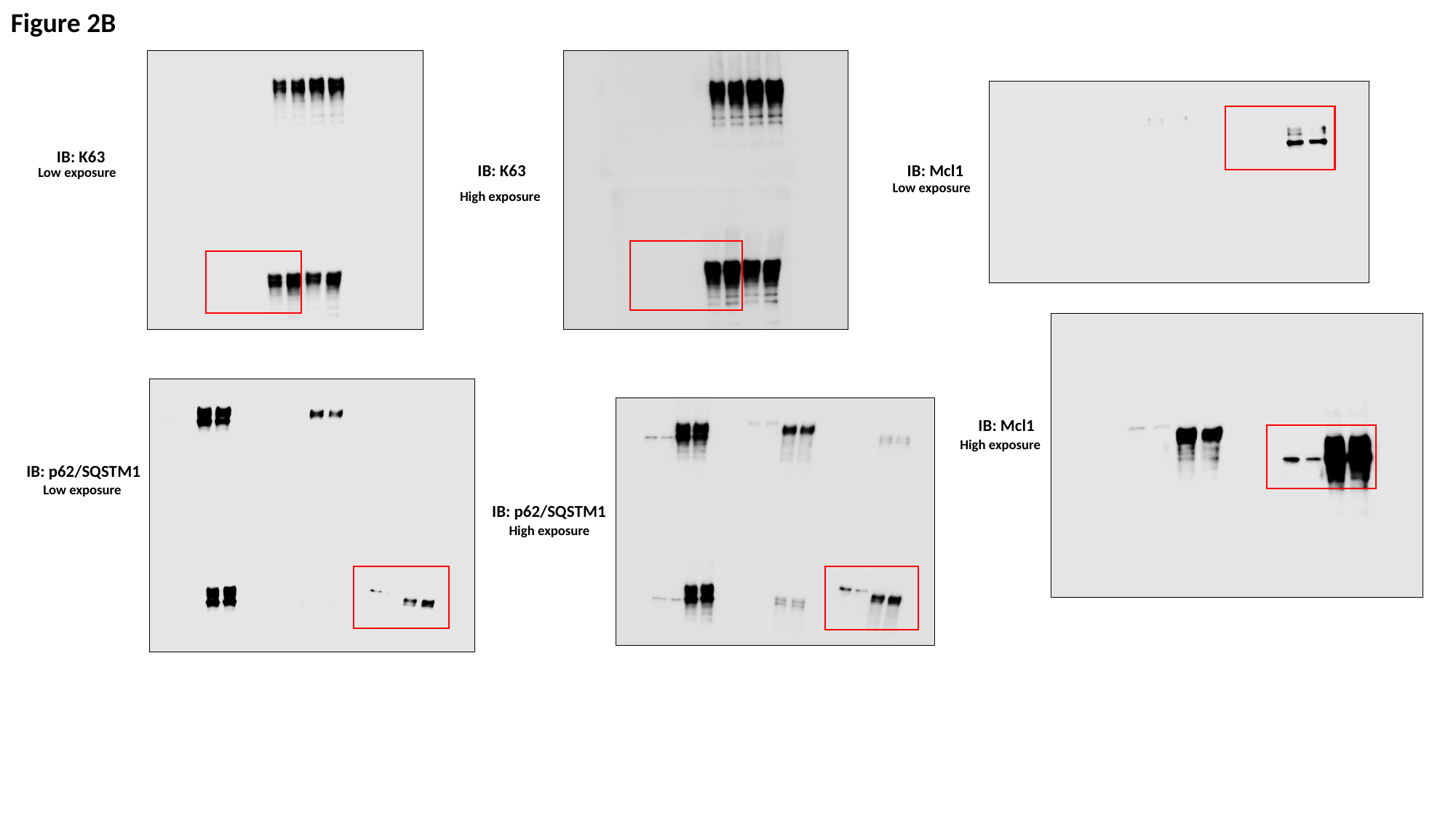

Figure 2B
IB: K63
IB: K63
IB: Mcl1
Low exposure
Low exposure
High exposure
IB: Mcl1
High exposure
IB: p62/SQSTM1
Low exposure
IB: p62/SQSTM1
High exposure

## Slide 10
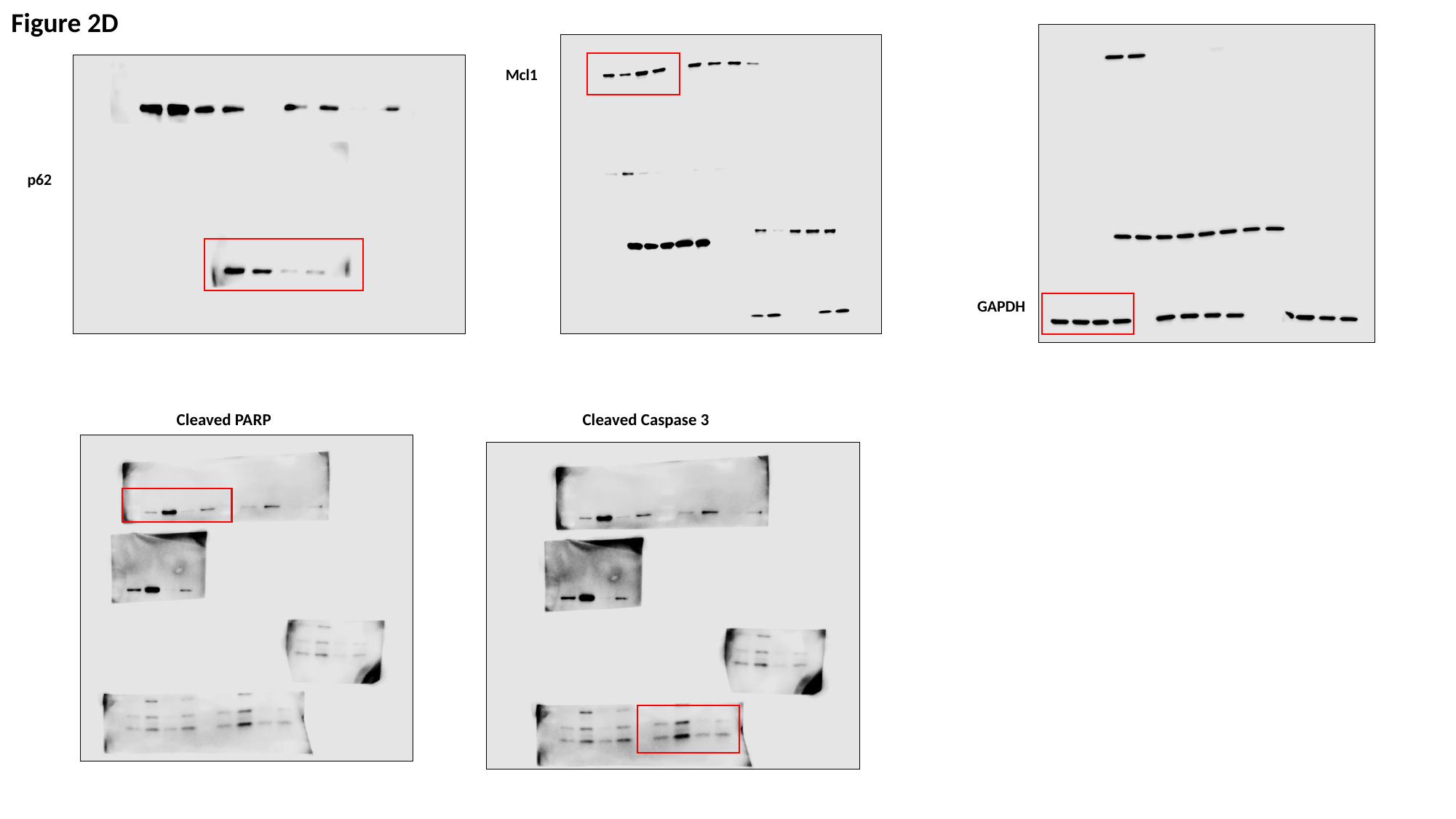

Figure 2D
Mcl1
p62
GAPDH
Cleaved PARP
Cleaved Caspase 3

## Slide 11
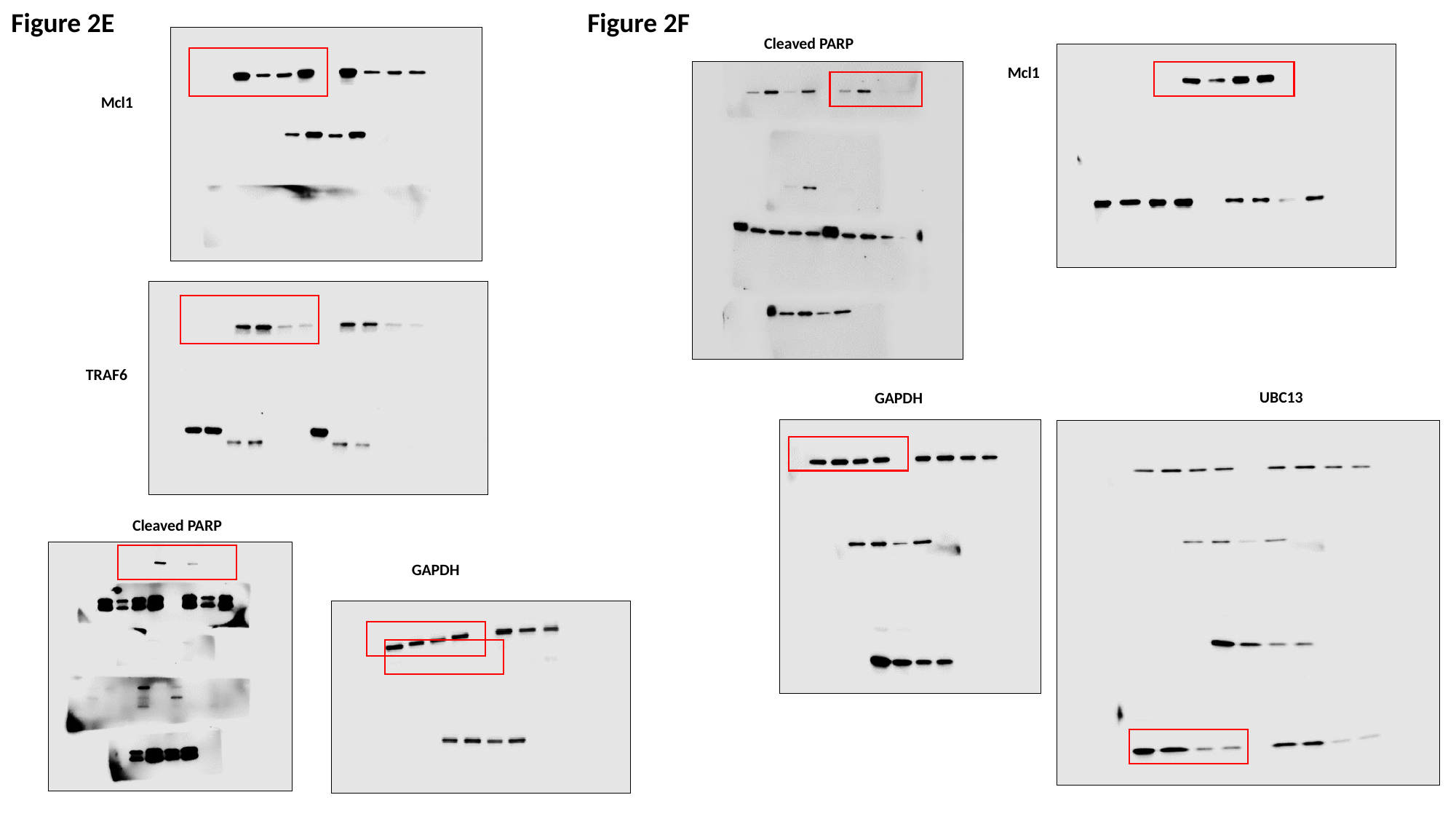

Figure 2E
Figure 2F
Cleaved PARP
Mcl1
Mcl1
TRAF6
UBC13
GAPDH
Cleaved PARP
GAPDH

## Slide 12
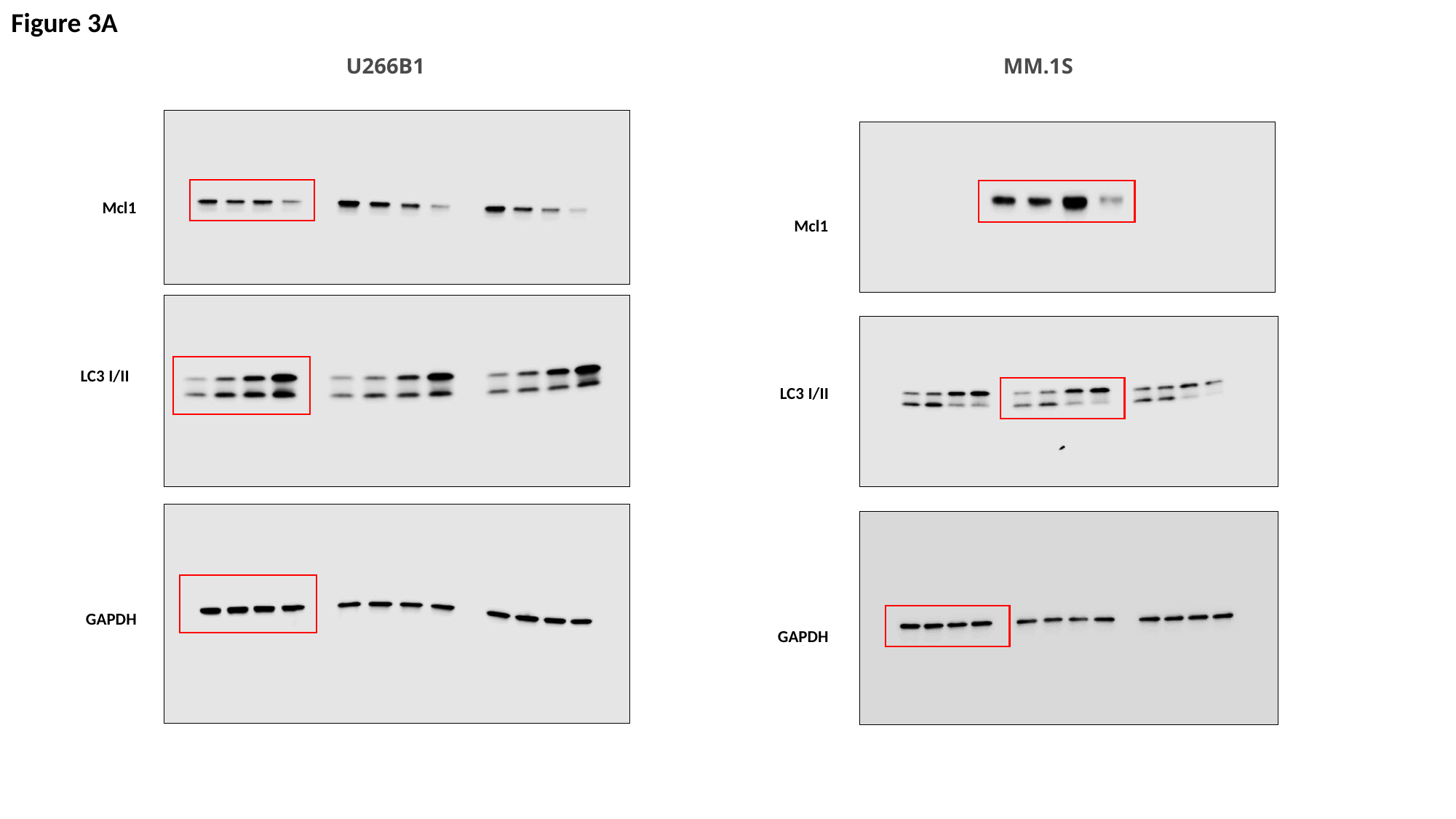

Figure 3A
U266B1
MM.1S
Mcl1
Mcl1
LC3 I/II
LC3 I/II
GAPDH
GAPDH

## Slide 13
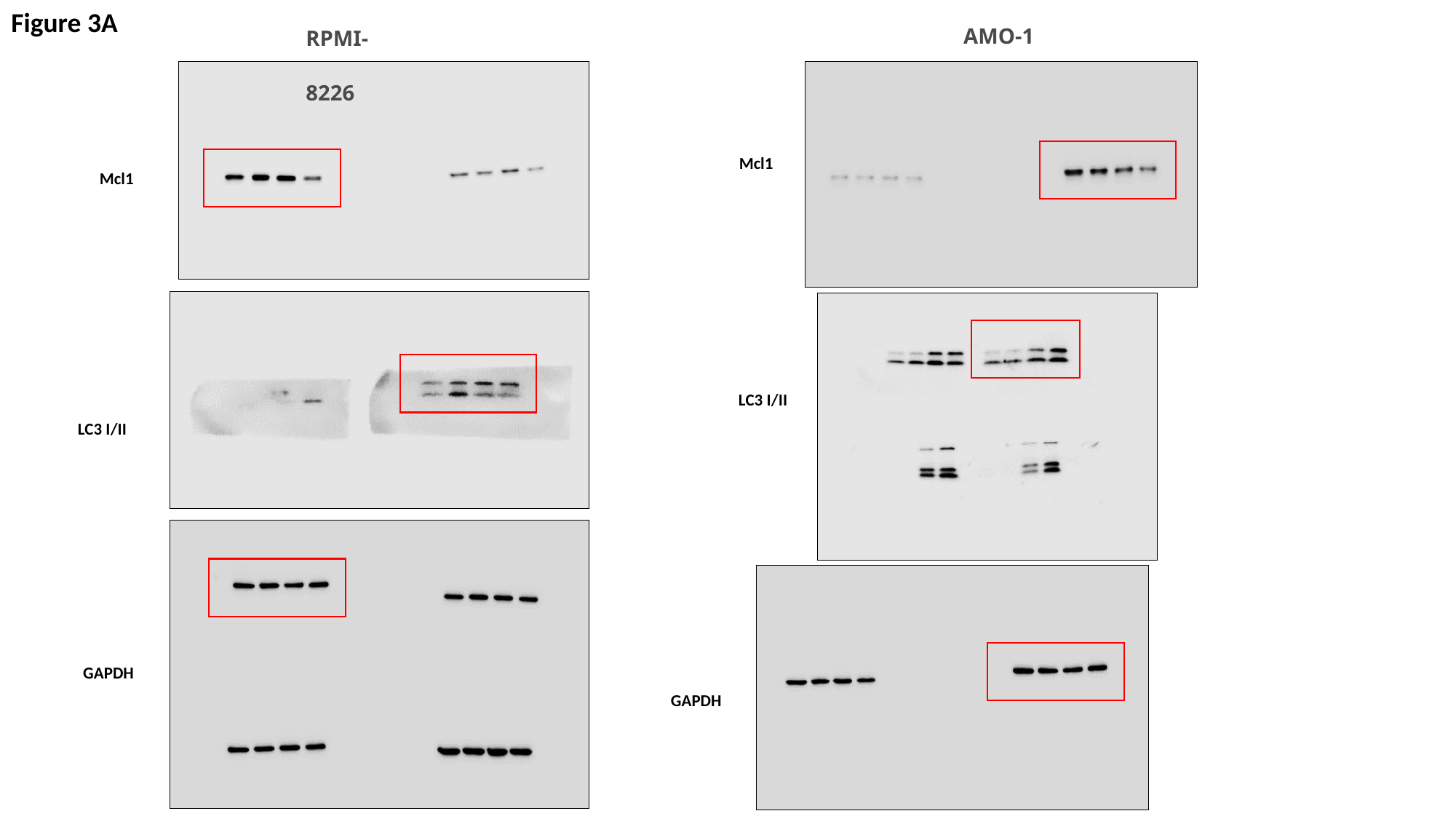

Figure 3A
AMO-1
RPMI-8226
Mcl1
Mcl1
LC3 I/II
LC3 I/II
GAPDH
GAPDH

## Slide 14
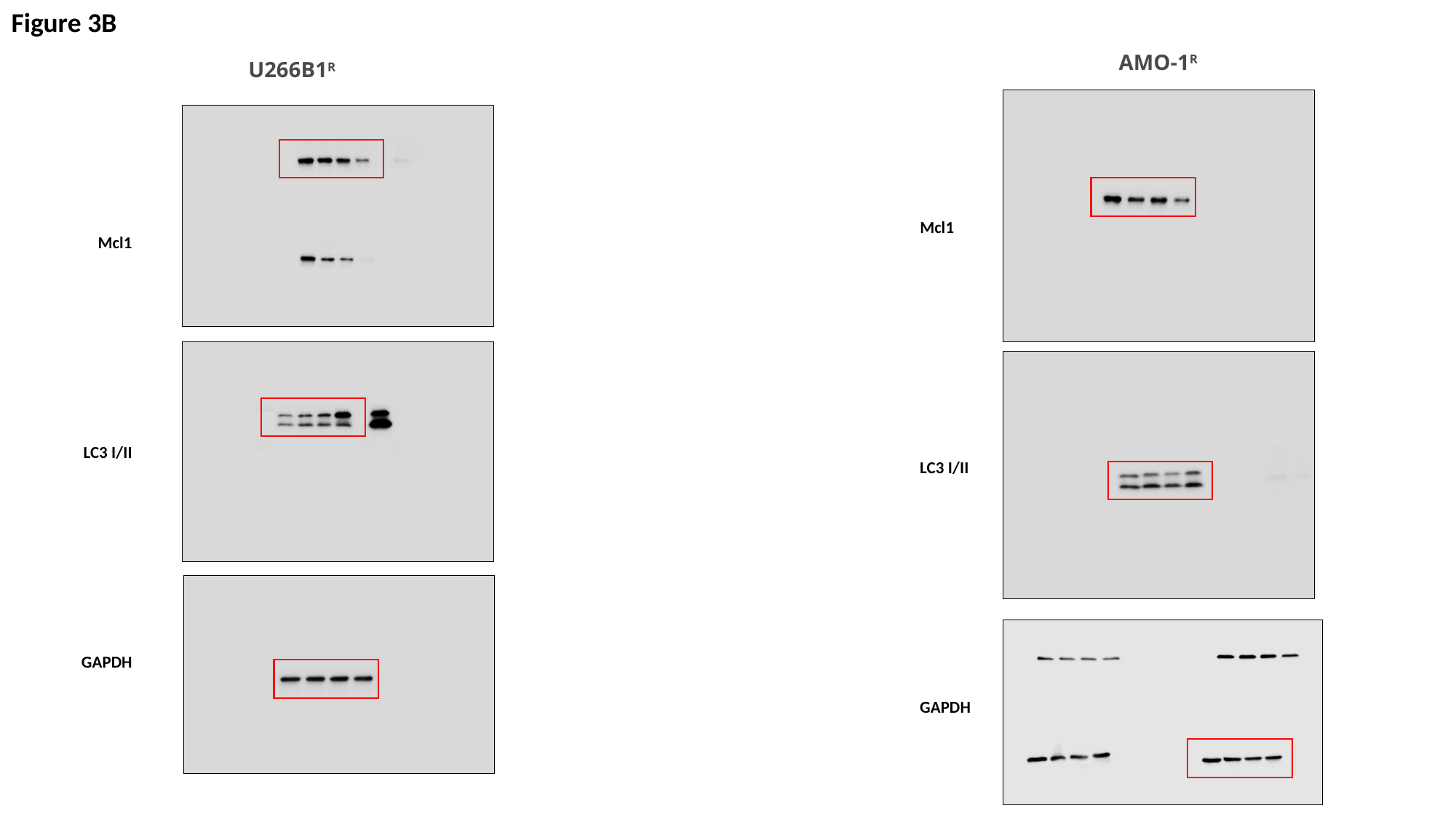

Figure 3B
AMO-1R
U266B1R
Mcl1
Mcl1
LC3 I/II
LC3 I/II
GAPDH
GAPDH

## Slide 15
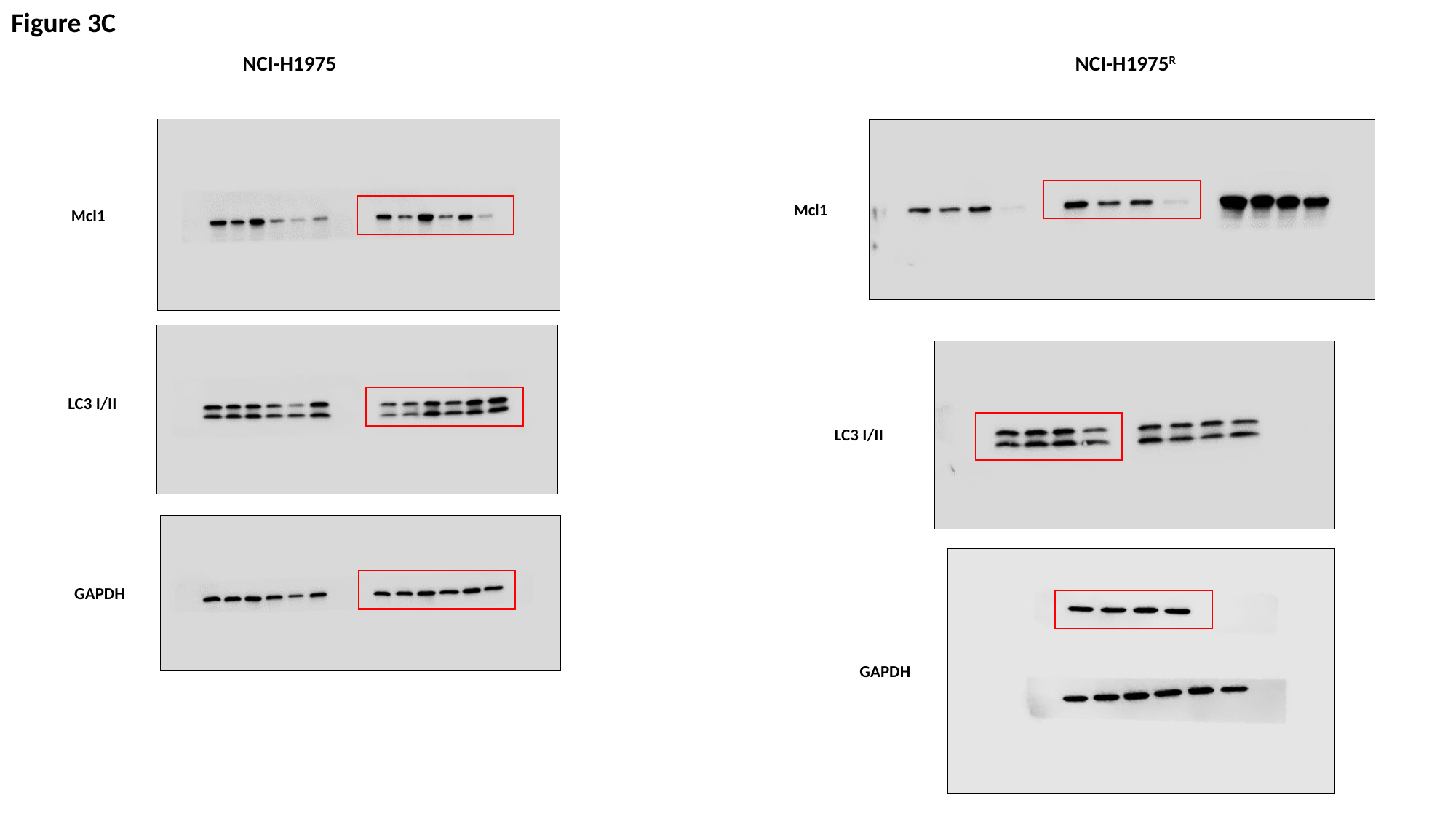

Figure 3C
NCI-H1975
NCI-H1975R
Mcl1
Mcl1
LC3 I/II
LC3 I/II
GAPDH
GAPDH

## Slide 16
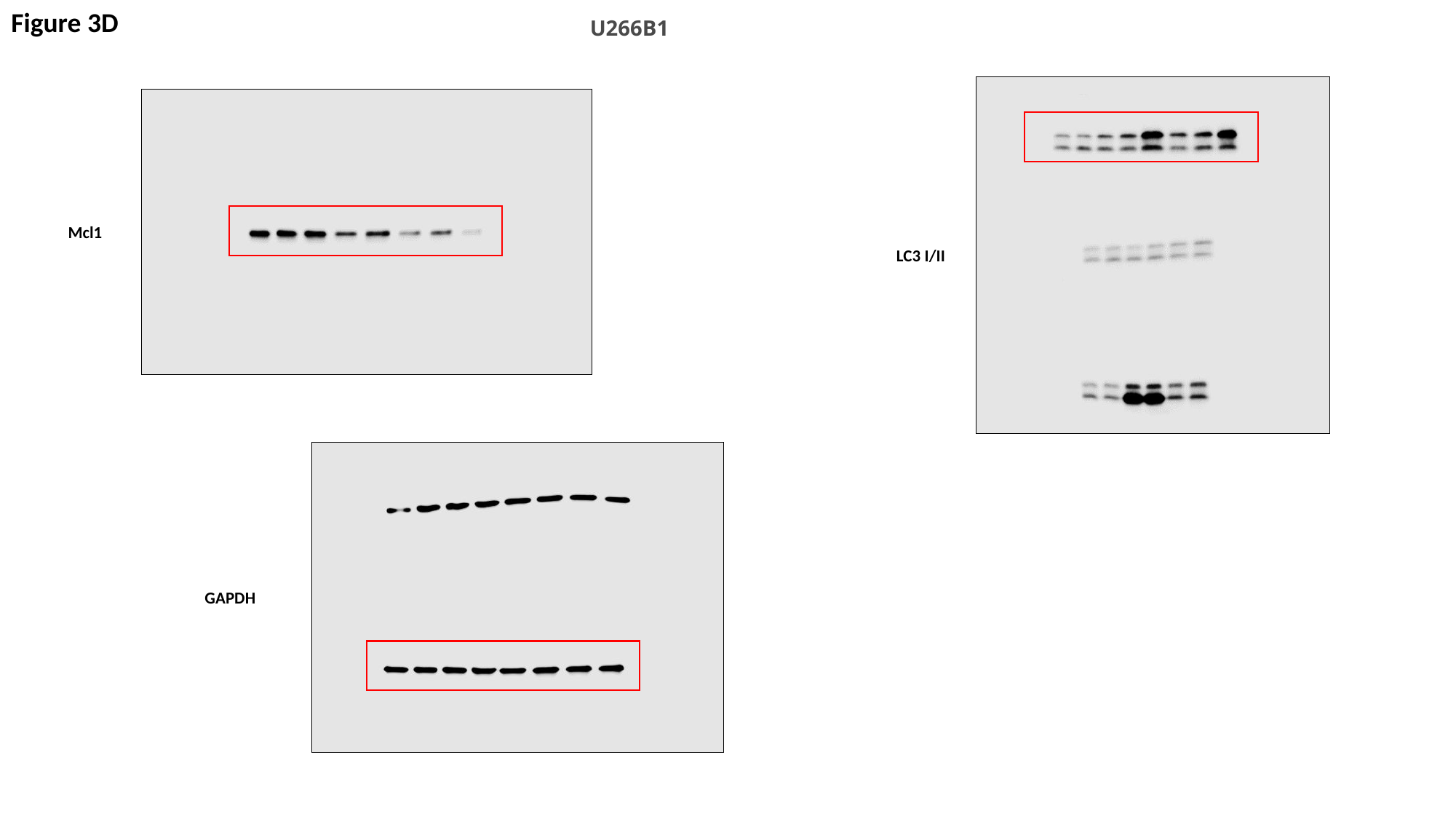

Figure 3D
U266B1
Mcl1
LC3 I/II
GAPDH

## Slide 17
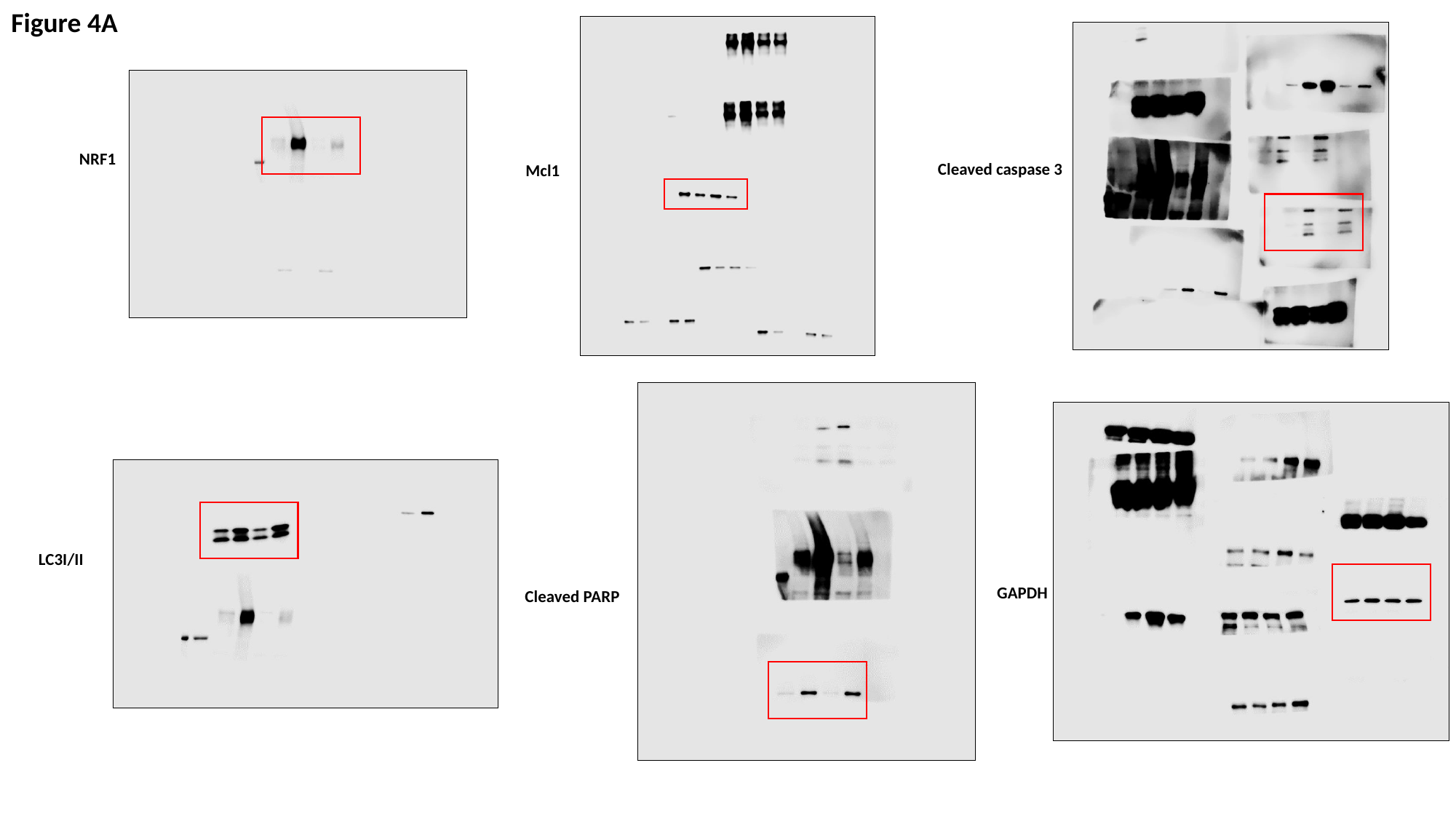

Figure 4A
NRF1
Cleaved caspase 3
Mcl1
LC3I/II
GAPDH
Cleaved PARP

## Slide 18
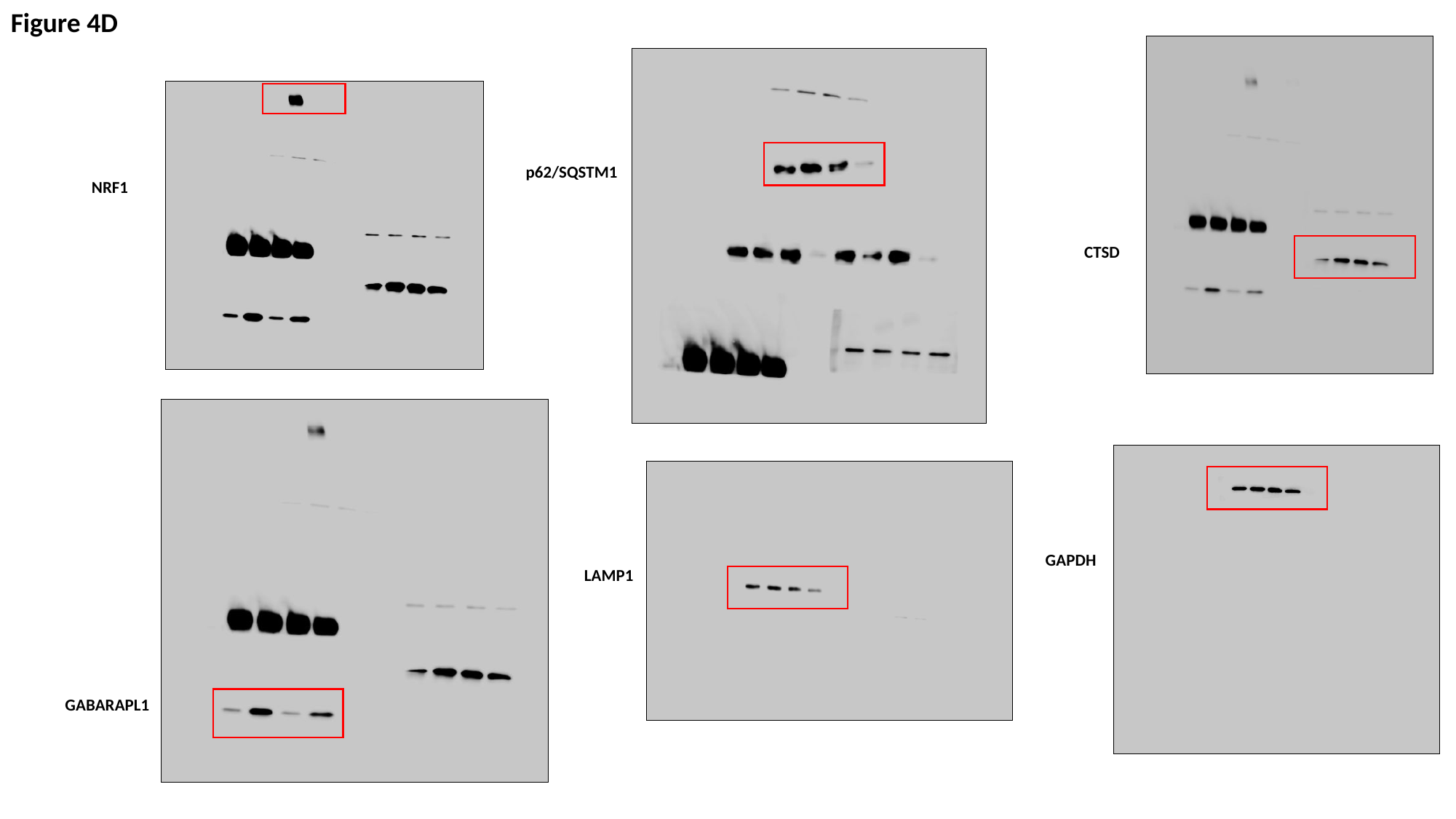

Figure 4D
p62/SQSTM1
NRF1
CTSD
GAPDH
LAMP1
GABARAPL1

## Slide 19
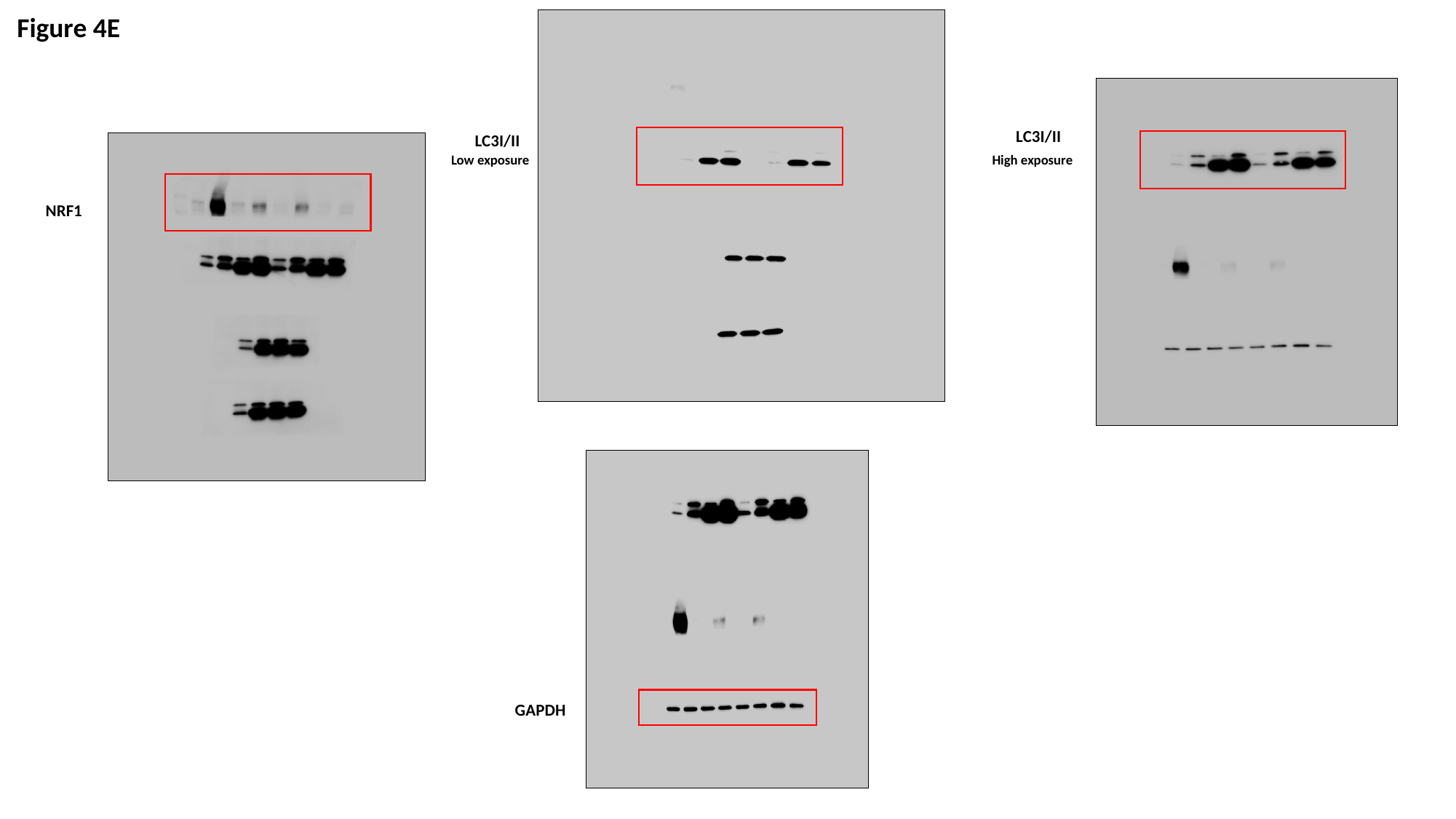

Figure 4E
LC3I/II
LC3I/II
Low exposure
High exposure
NRF1
GAPDH

## Slide 20
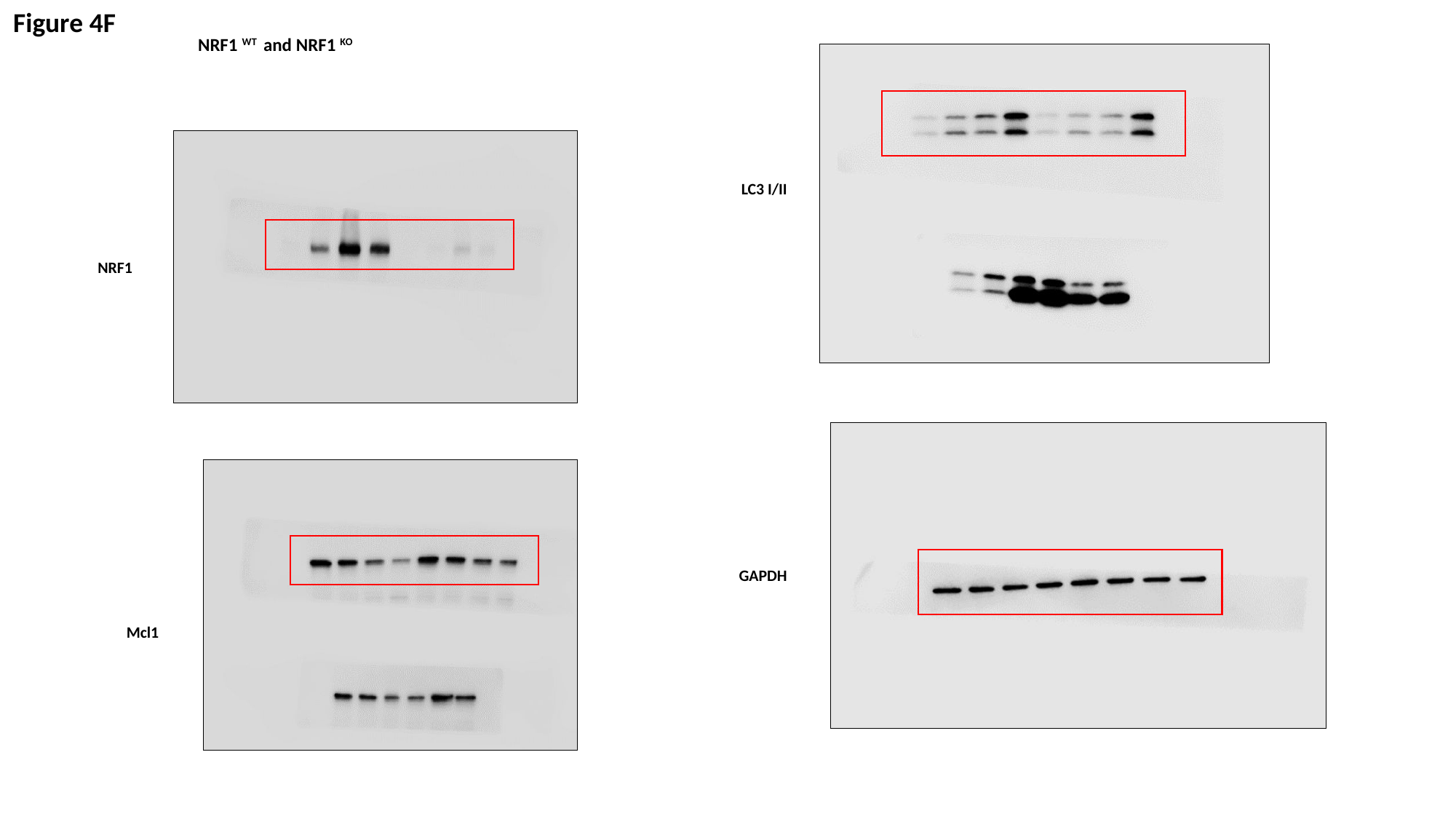

Figure 4F
NRF1 WT
and NRF1 KO
LC3 I/II
NRF1
GAPDH
Mcl1

## Slide 21
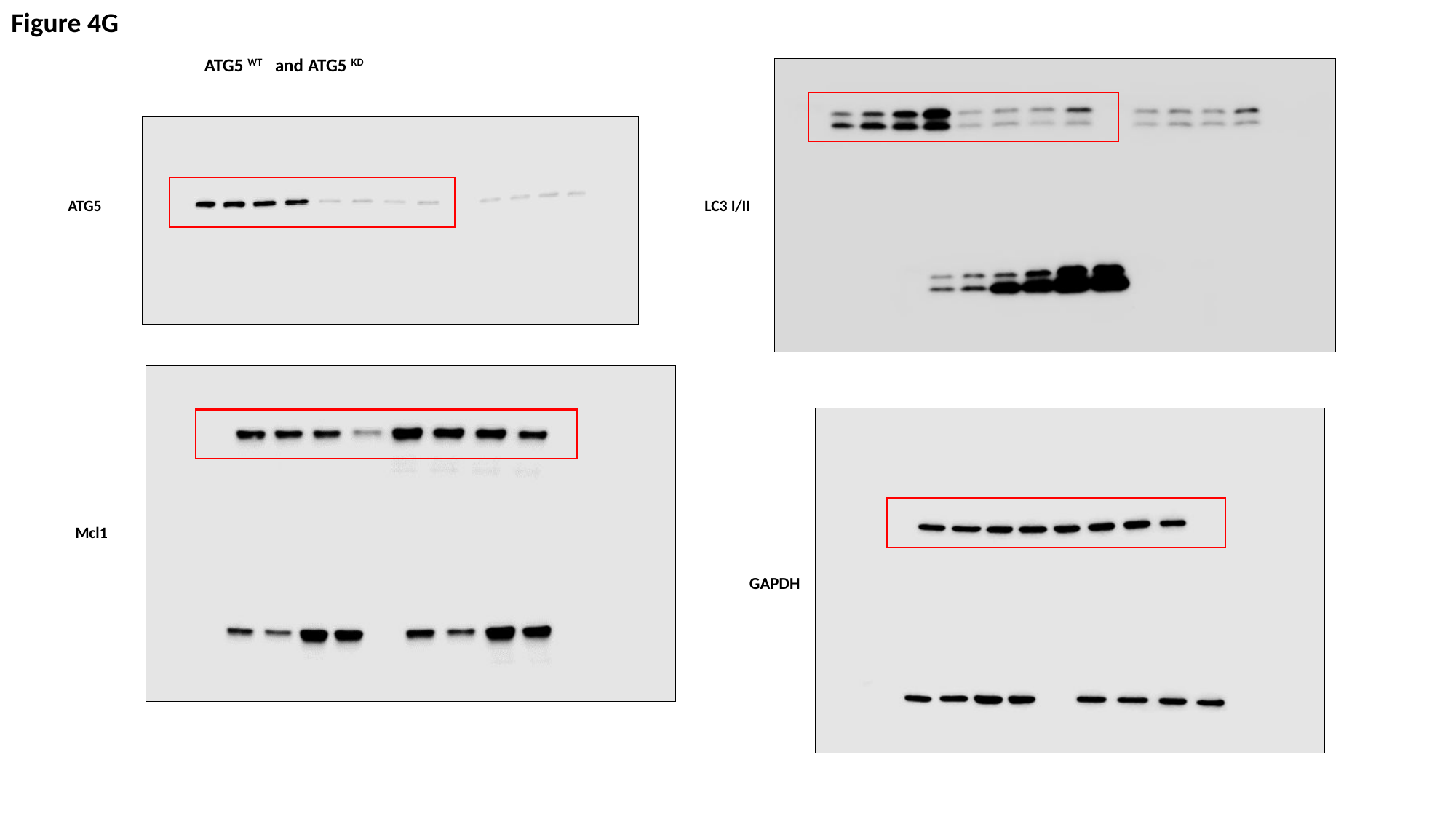

Figure 4G
ATG5 WT
and ATG5 KD
ATG5
LC3 I/II
Mcl1
GAPDH

## Slide 22
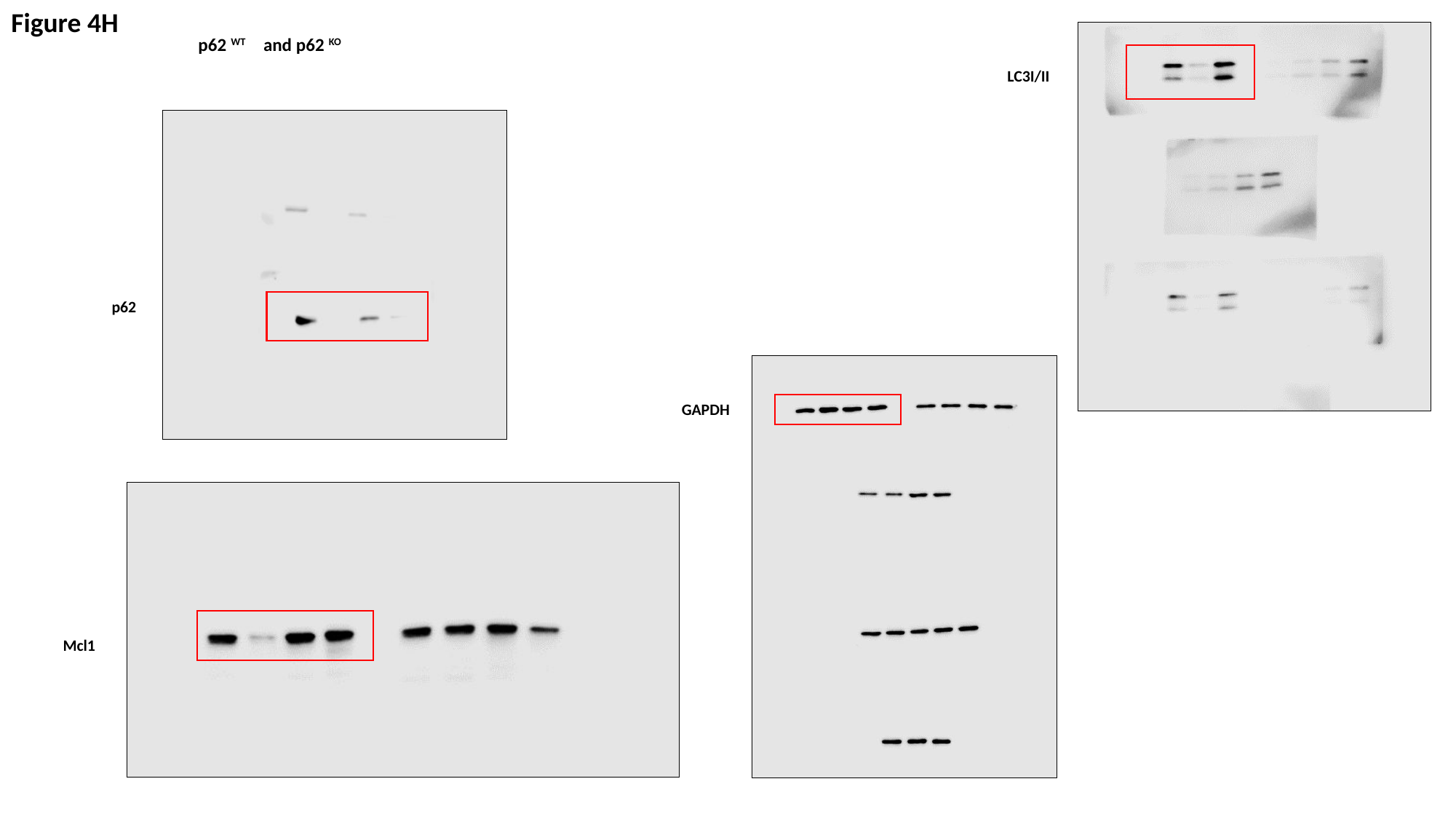

Figure 4H
p62 WT
and p62 KO
LC3I/II
p62
GAPDH
Mcl1

## Slide 23
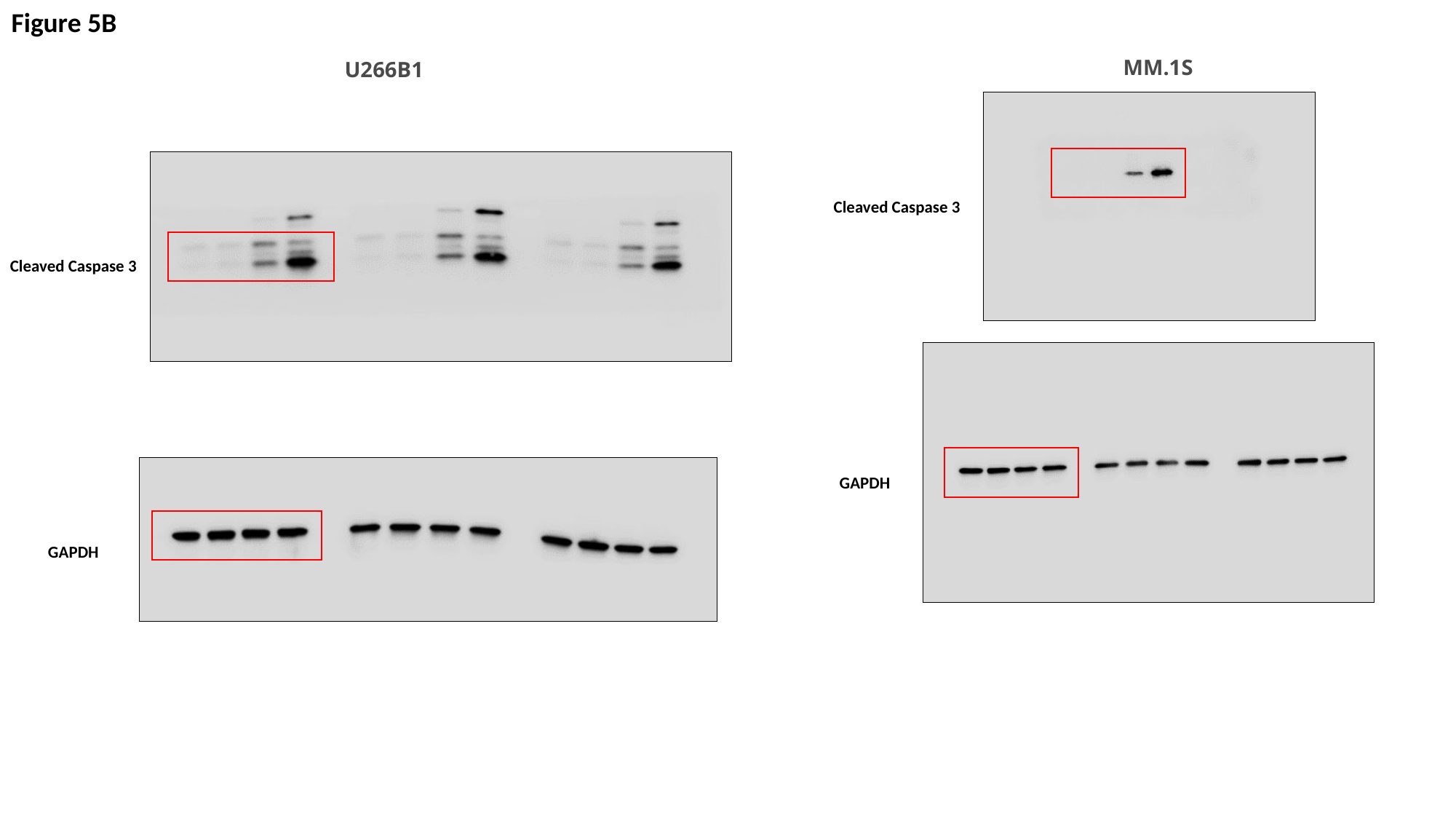

Figure 5B
MM.1S
U266B1
Cleaved Caspase 3
Cleaved Caspase 3
GAPDH
GAPDH

## Slide 24
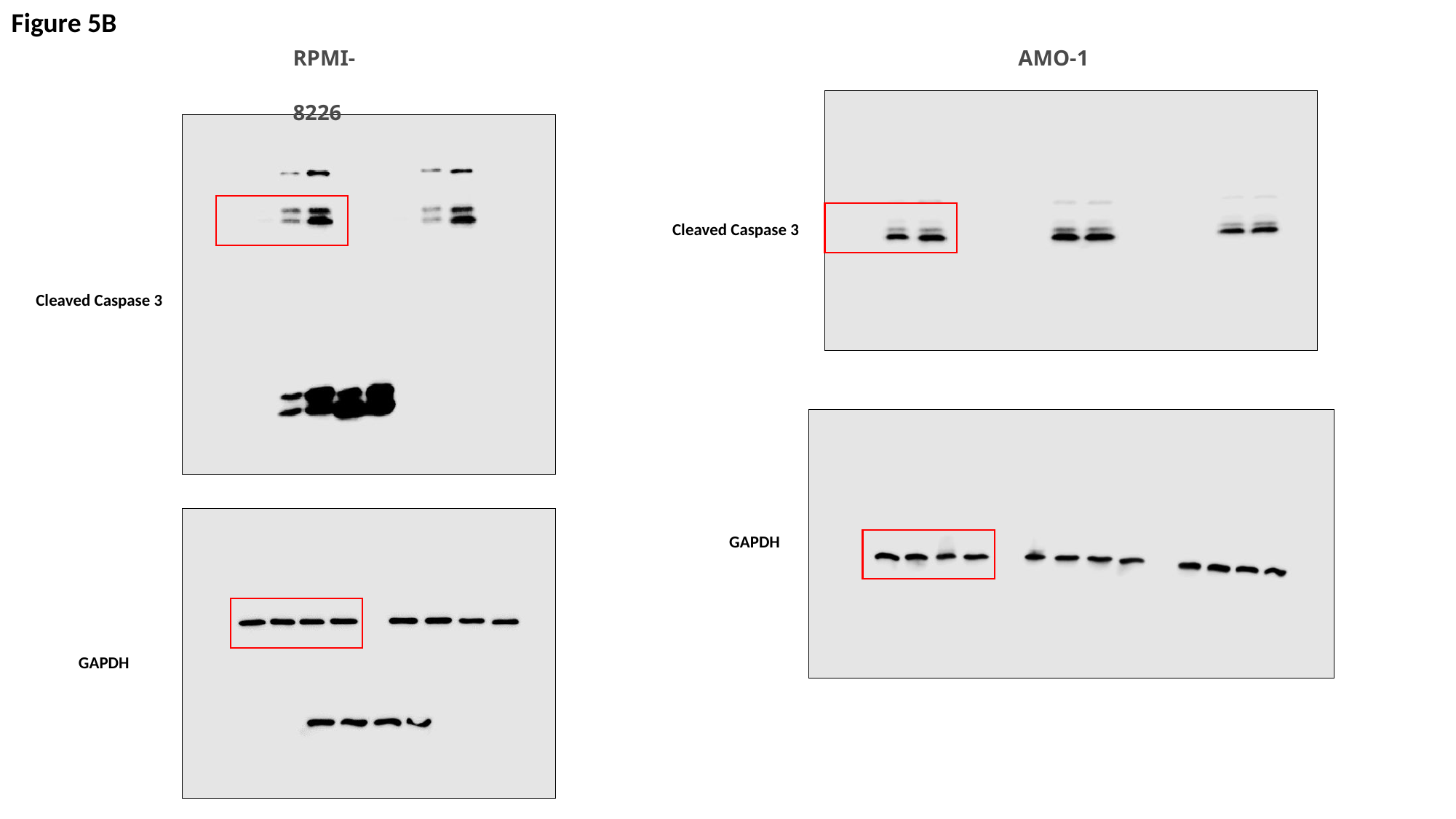

Figure 5B
RPMI-8226
AMO-1
Cleaved Caspase 3
Cleaved Caspase 3
GAPDH
GAPDH

## Slide 25
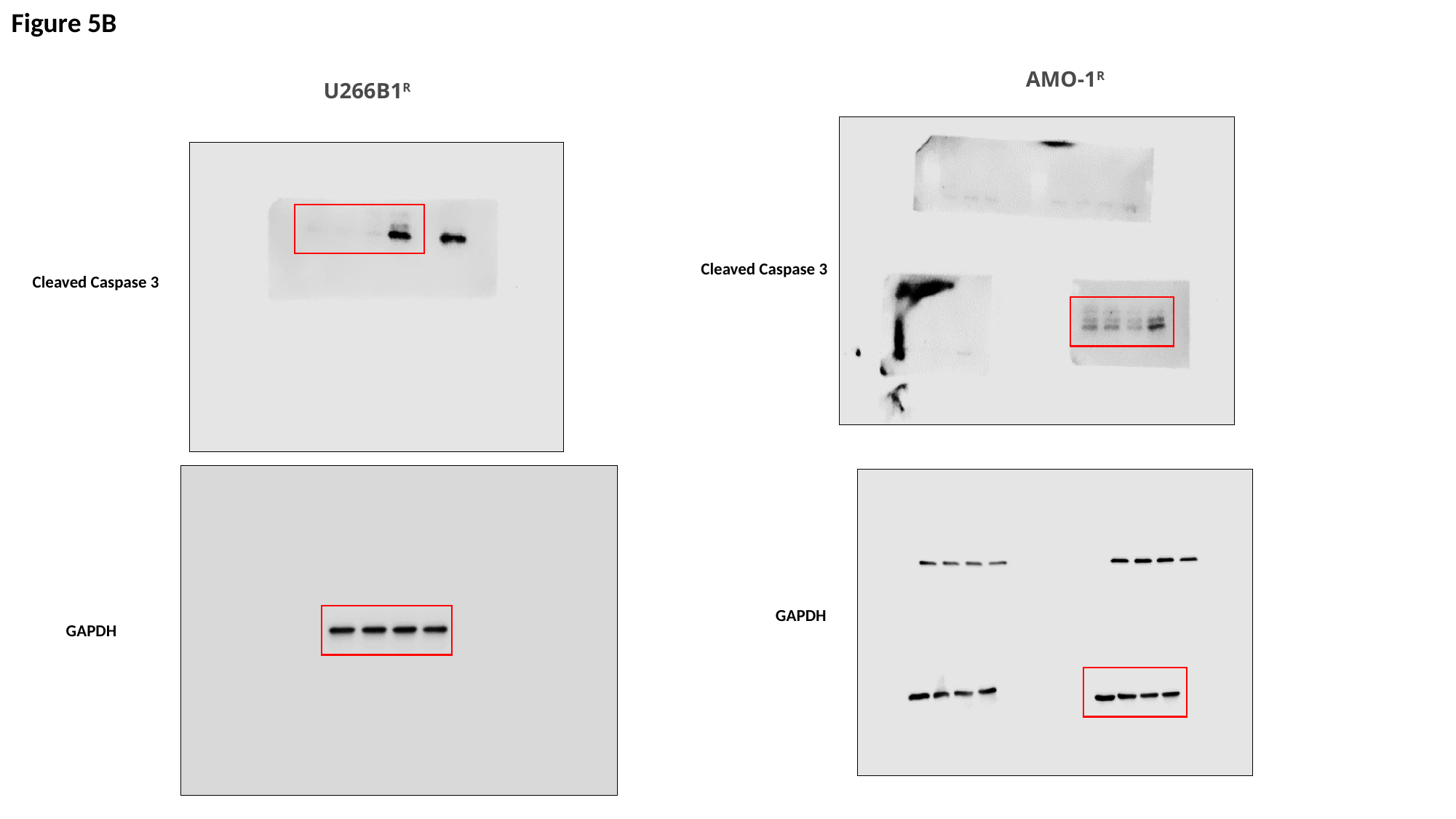

Figure 5B
AMO-1R
U266B1R
Cleaved Caspase 3
Cleaved Caspase 3
GAPDH
GAPDH

## Slide 26
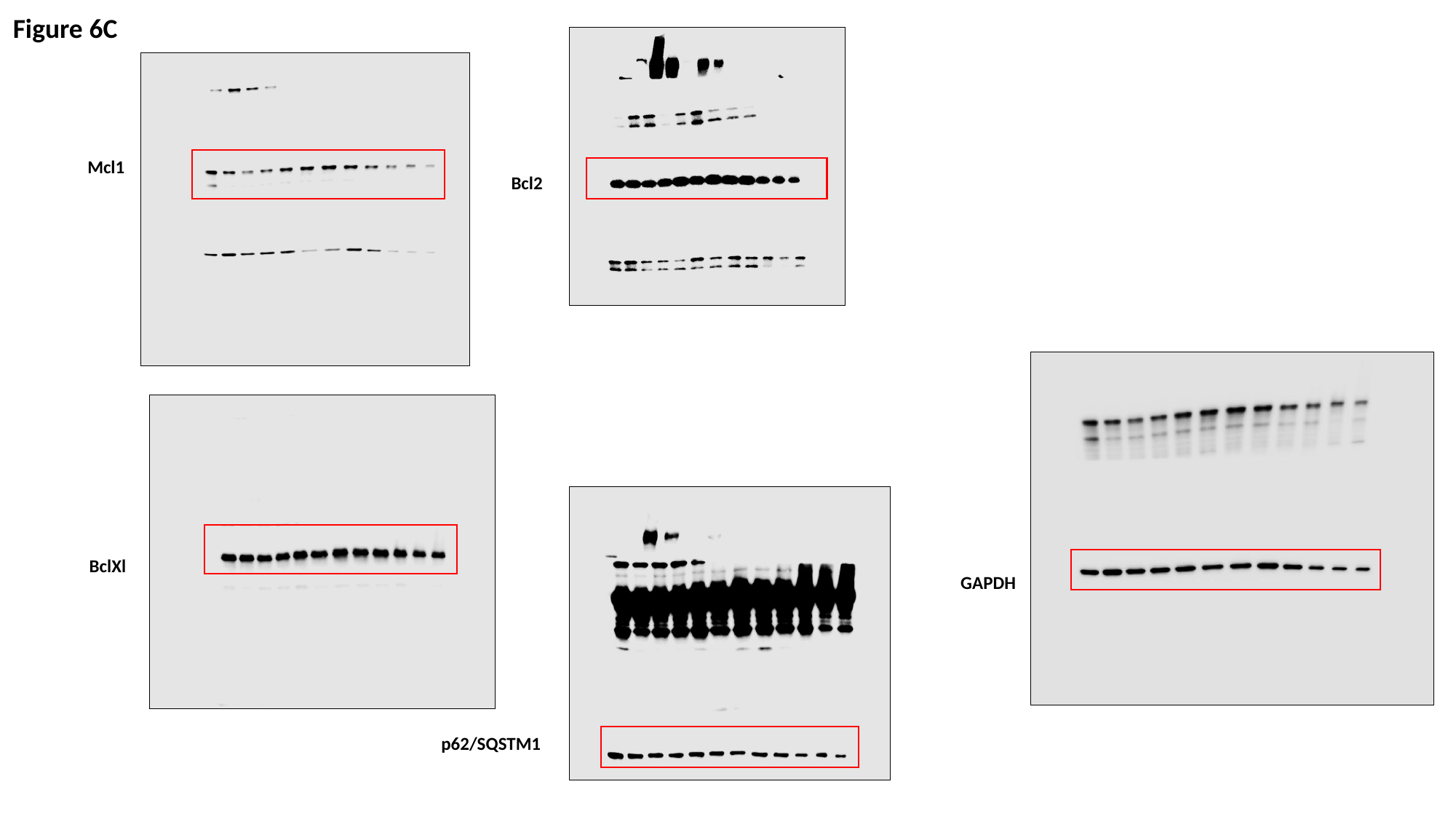

Figure 6C
Mcl1
Bcl2
BclXl
GAPDH
p62/SQSTM1

## Slide 27
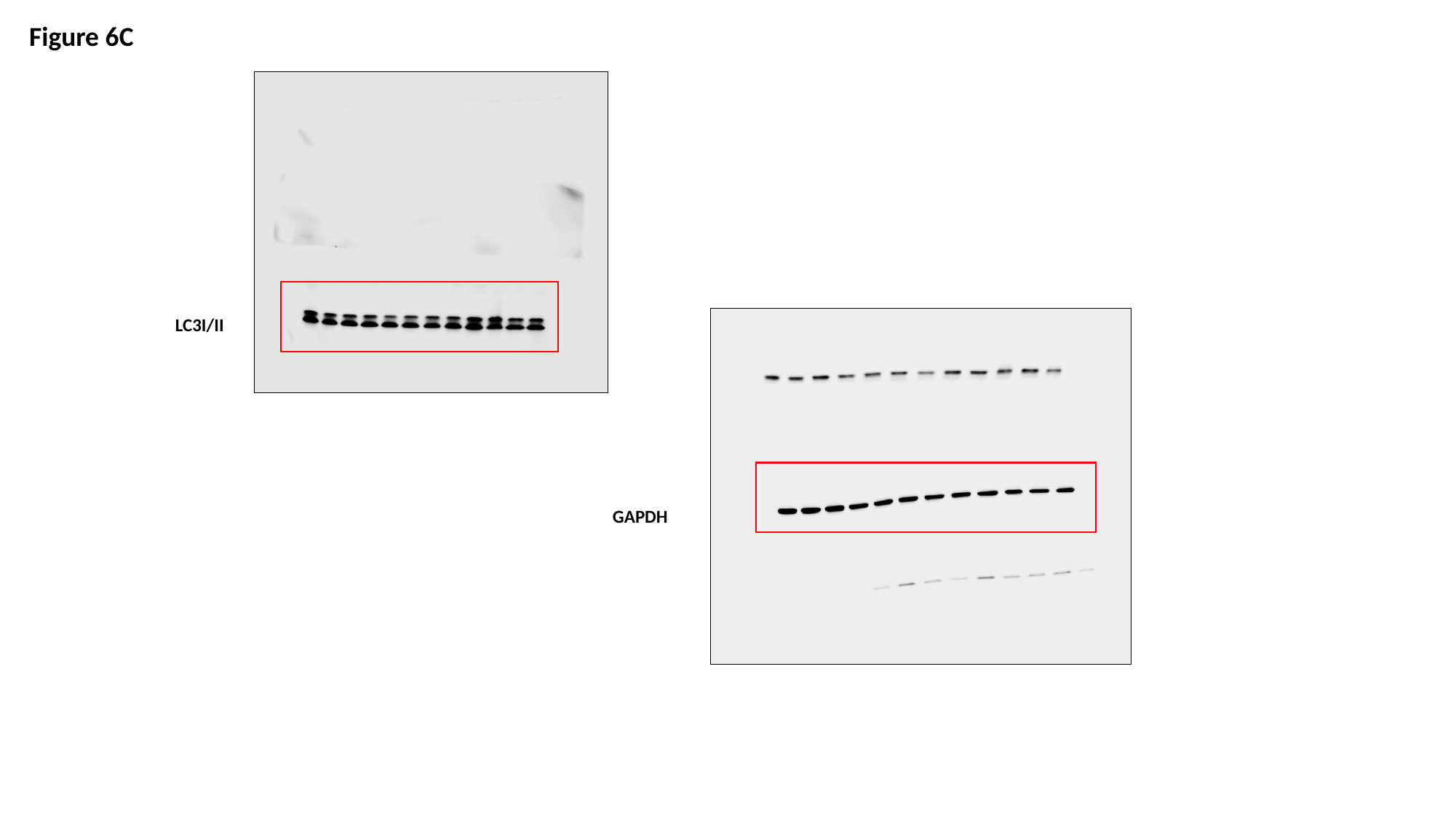

Figure 6C
LC3I/II
GAPDH

## Slide 28
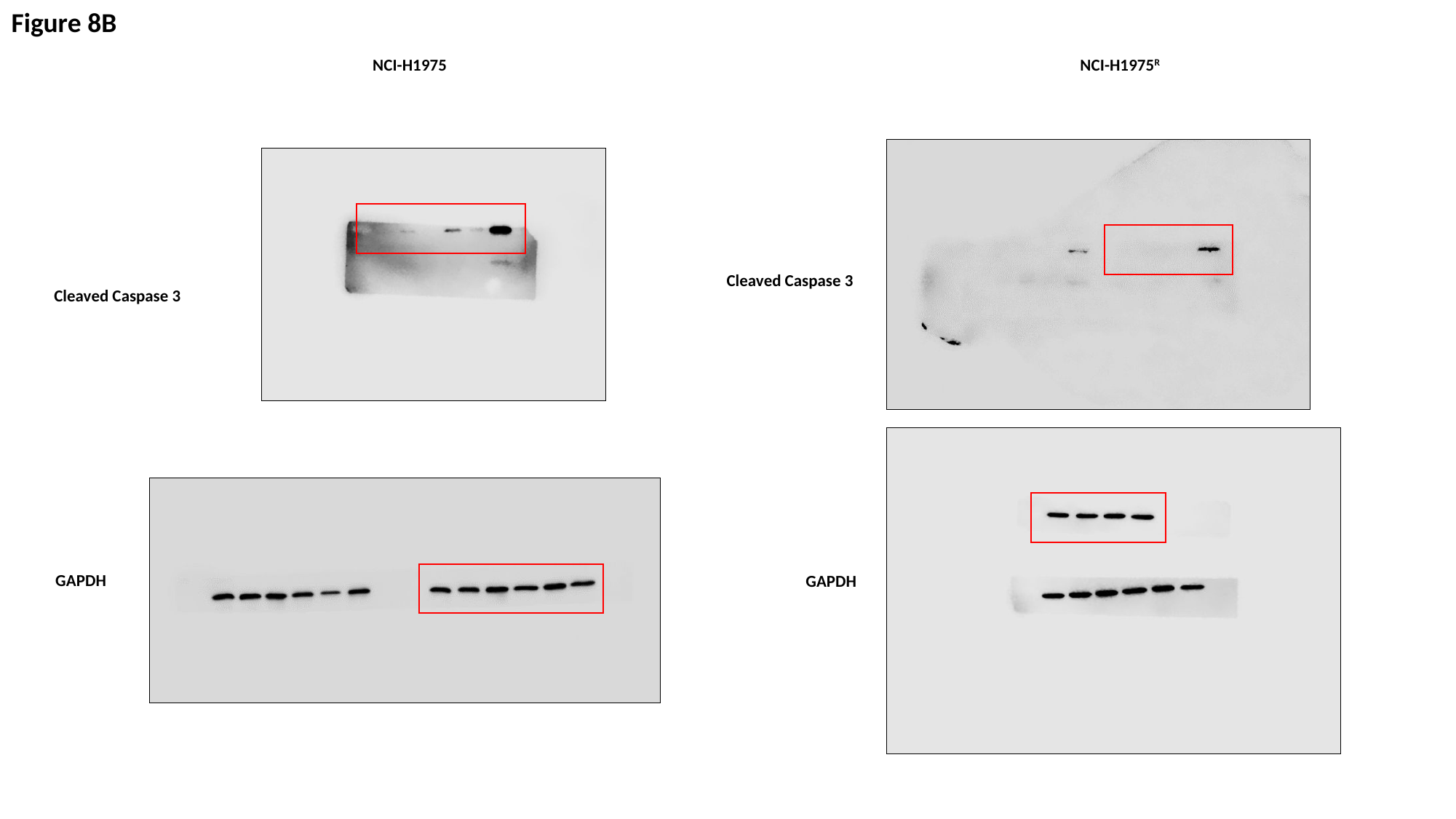

Figure 8B
NCI-H1975
NCI-H1975R
Cleaved Caspase 3
Cleaved Caspase 3
GAPDH
GAPDH

## Slide 29
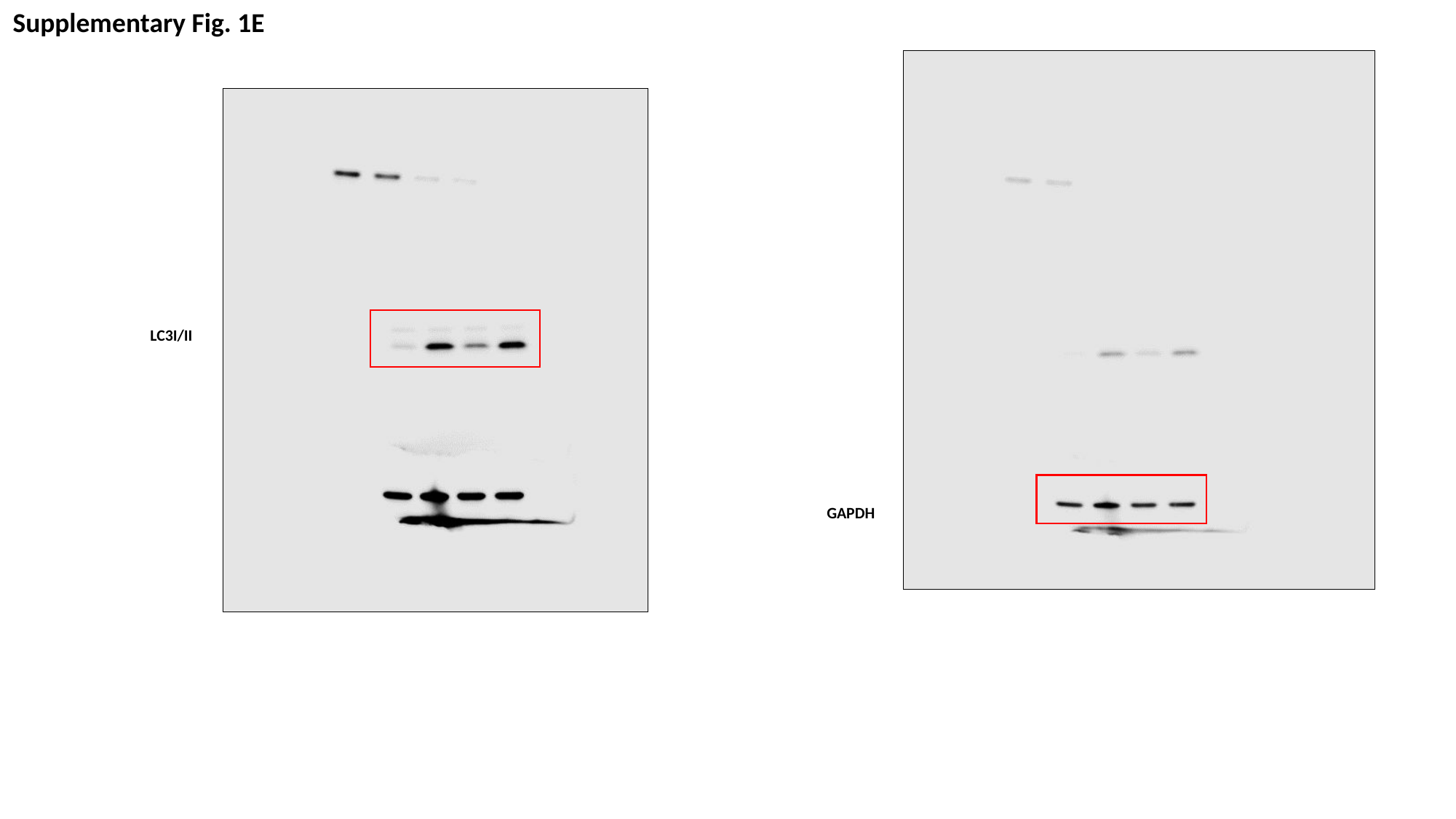

Supplementary Fig. 1E
LC3I/II
GAPDH

## Slide 30
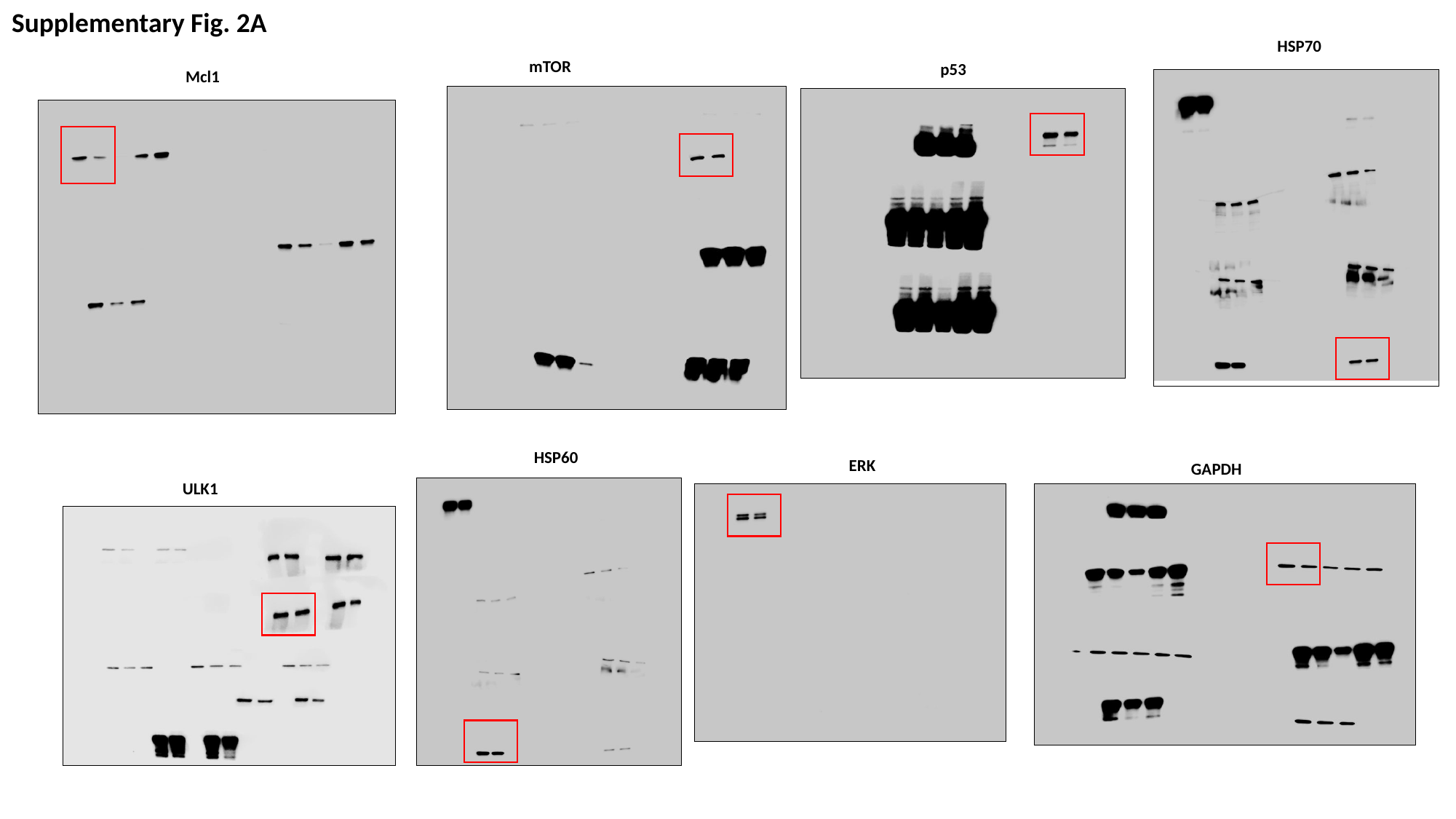

Supplementary Fig. 2A
HSP70
mTOR
p53
Mcl1
HSP60
ERK
GAPDH
ULK1

## Slide 31
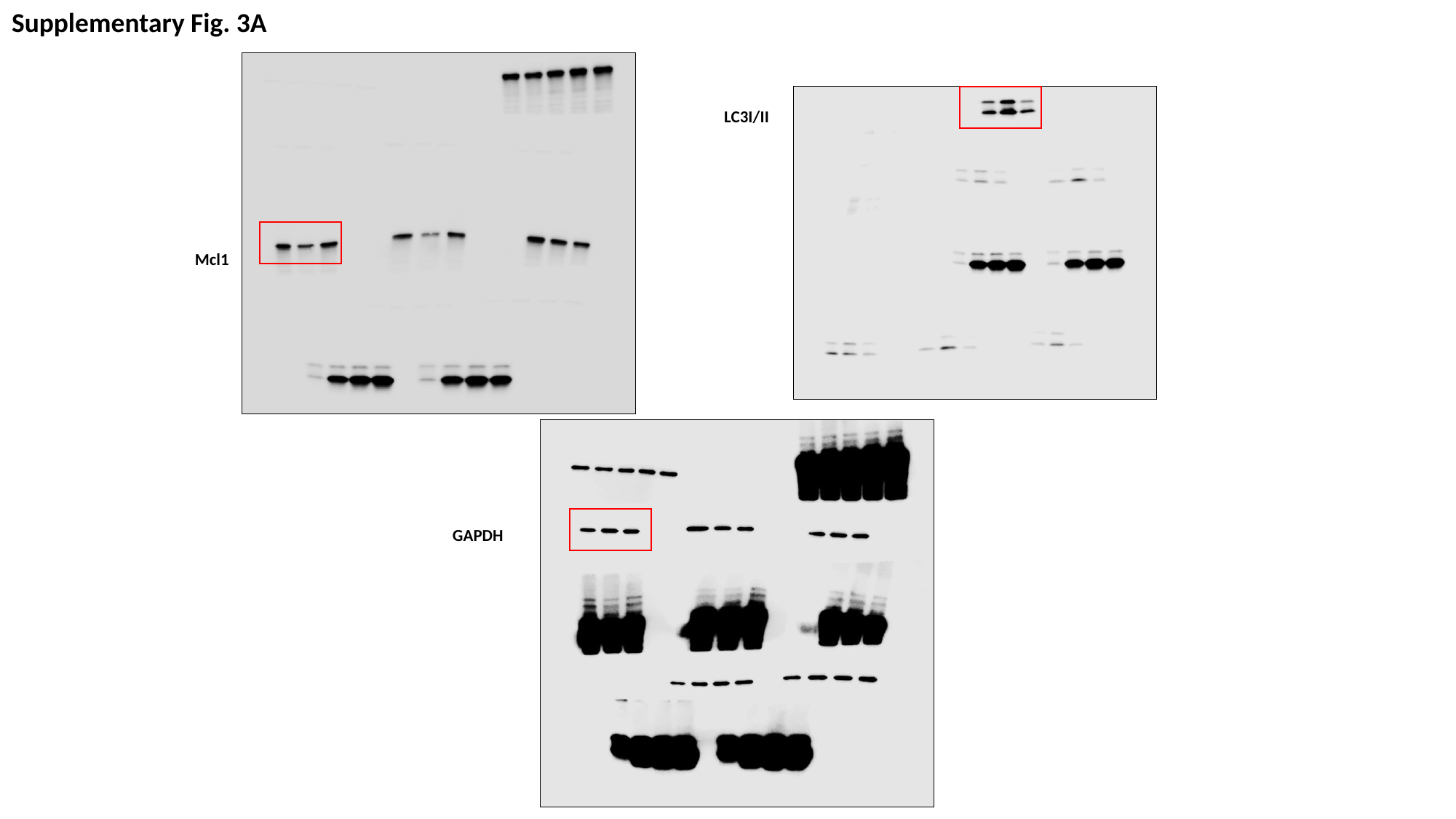

Supplementary Fig. 3A
LC3I/II
Mcl1
GAPDH

## Slide 32
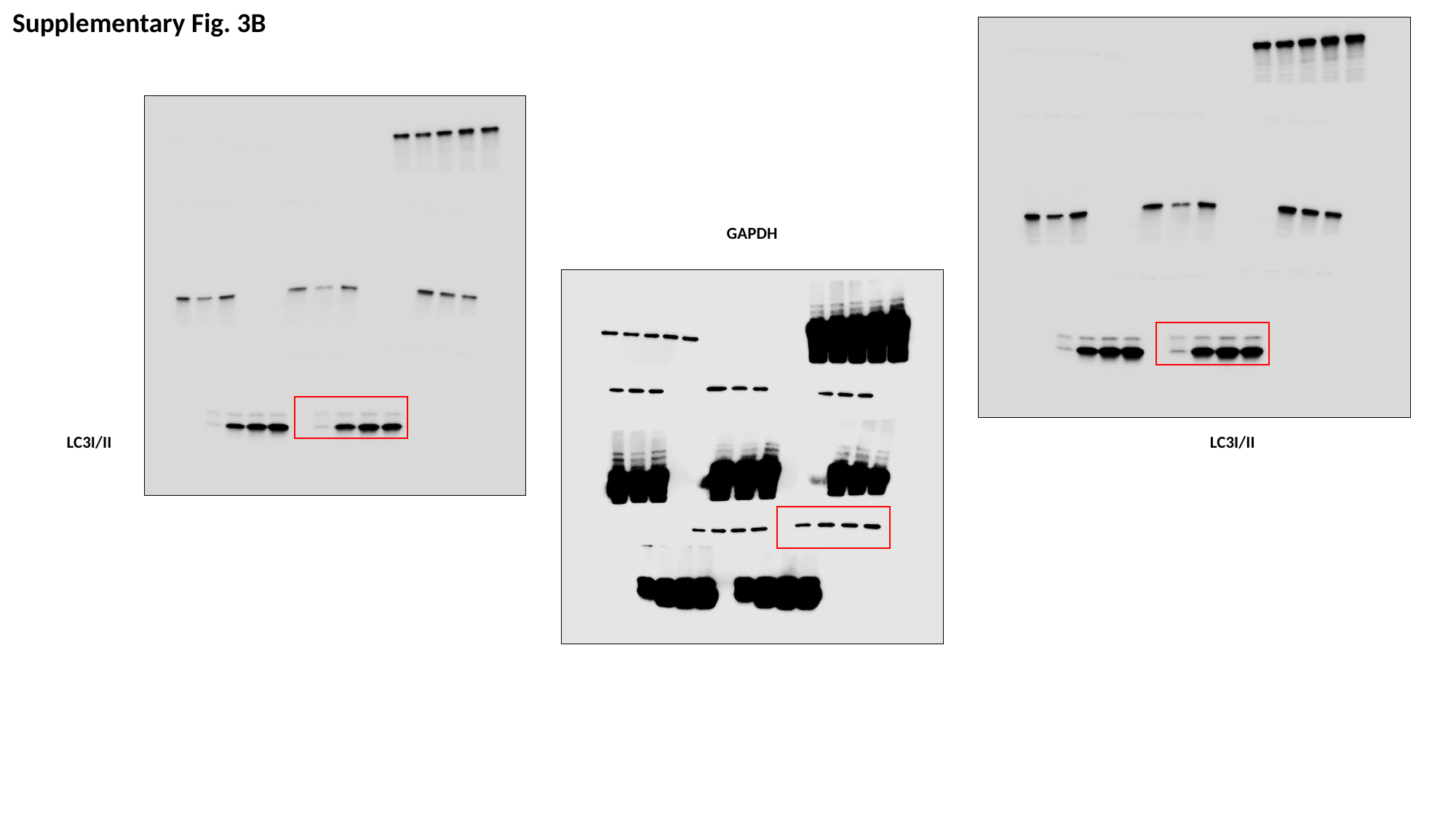

Supplementary Fig. 3B
GAPDH
LC3I/II
LC3I/II

## Slide 33
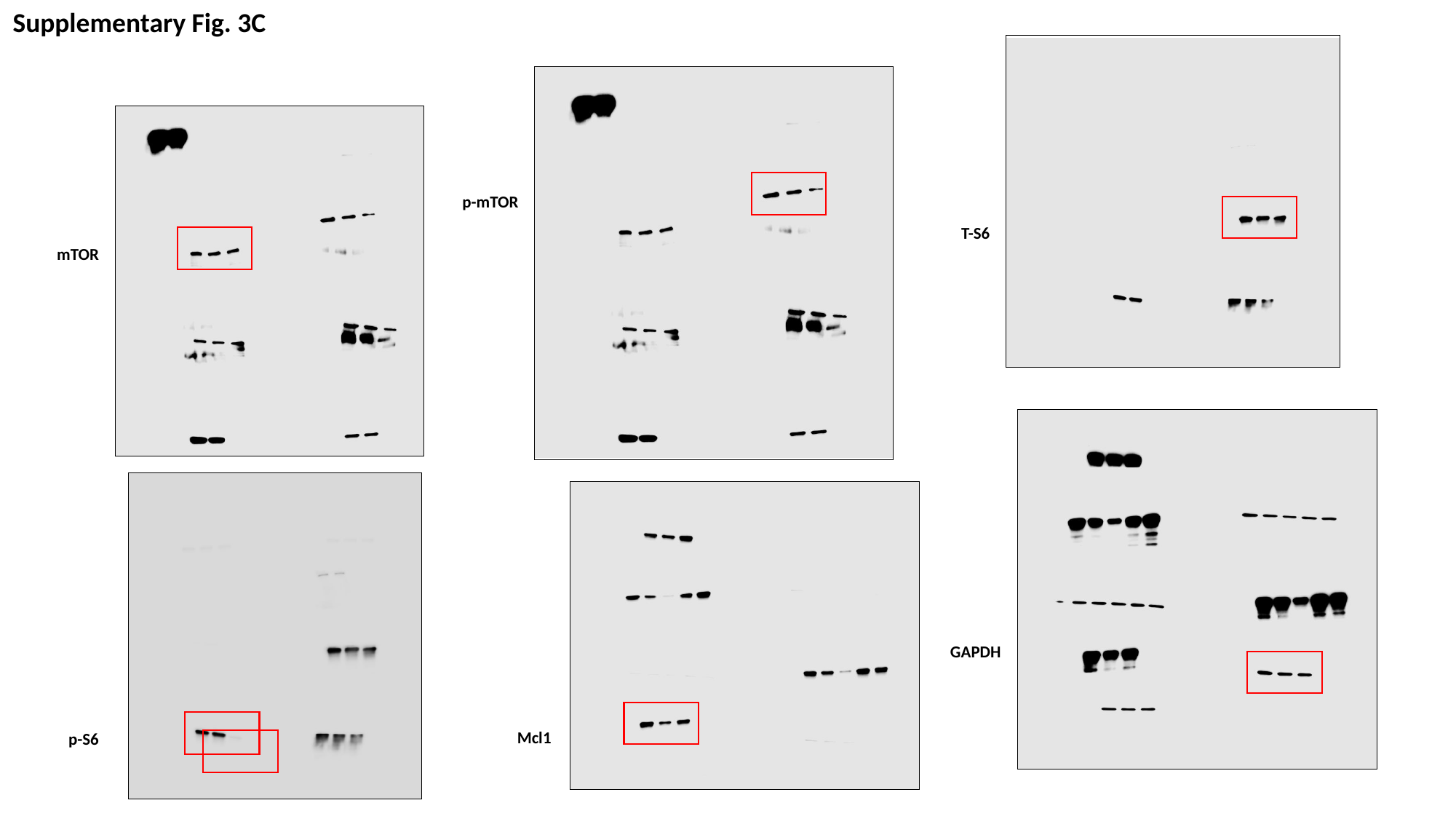

Supplementary Fig. 3C
p-mTOR
T-S6
mTOR
GAPDH
Mcl1
p-S6

## Slide 34
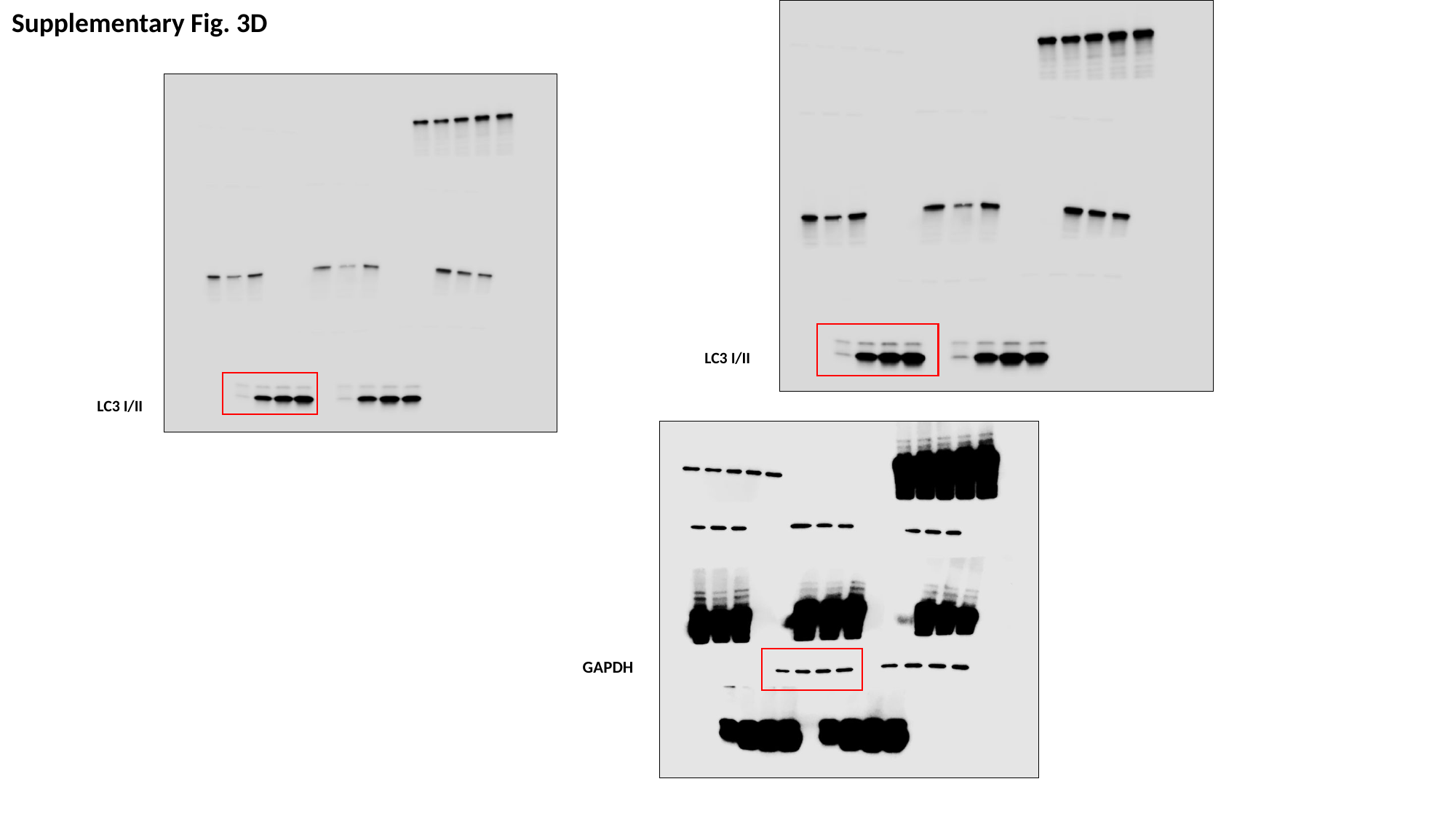

Supplementary Fig. 3D
LC3 I/II
LC3 I/II
GAPDH

## Slide 35
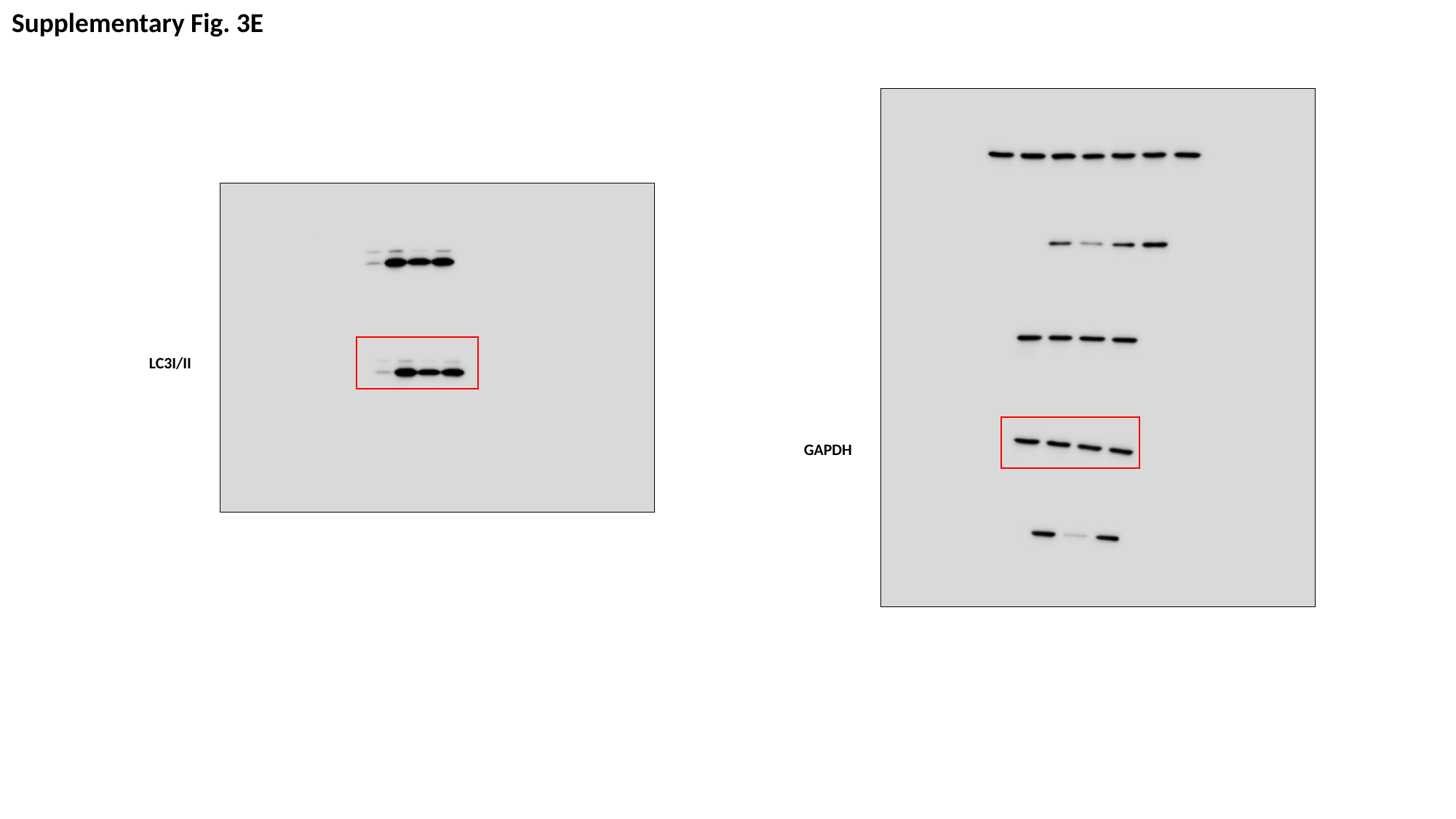

Supplementary Fig. 3E
LC3I/II
GAPDH

## Slide 36
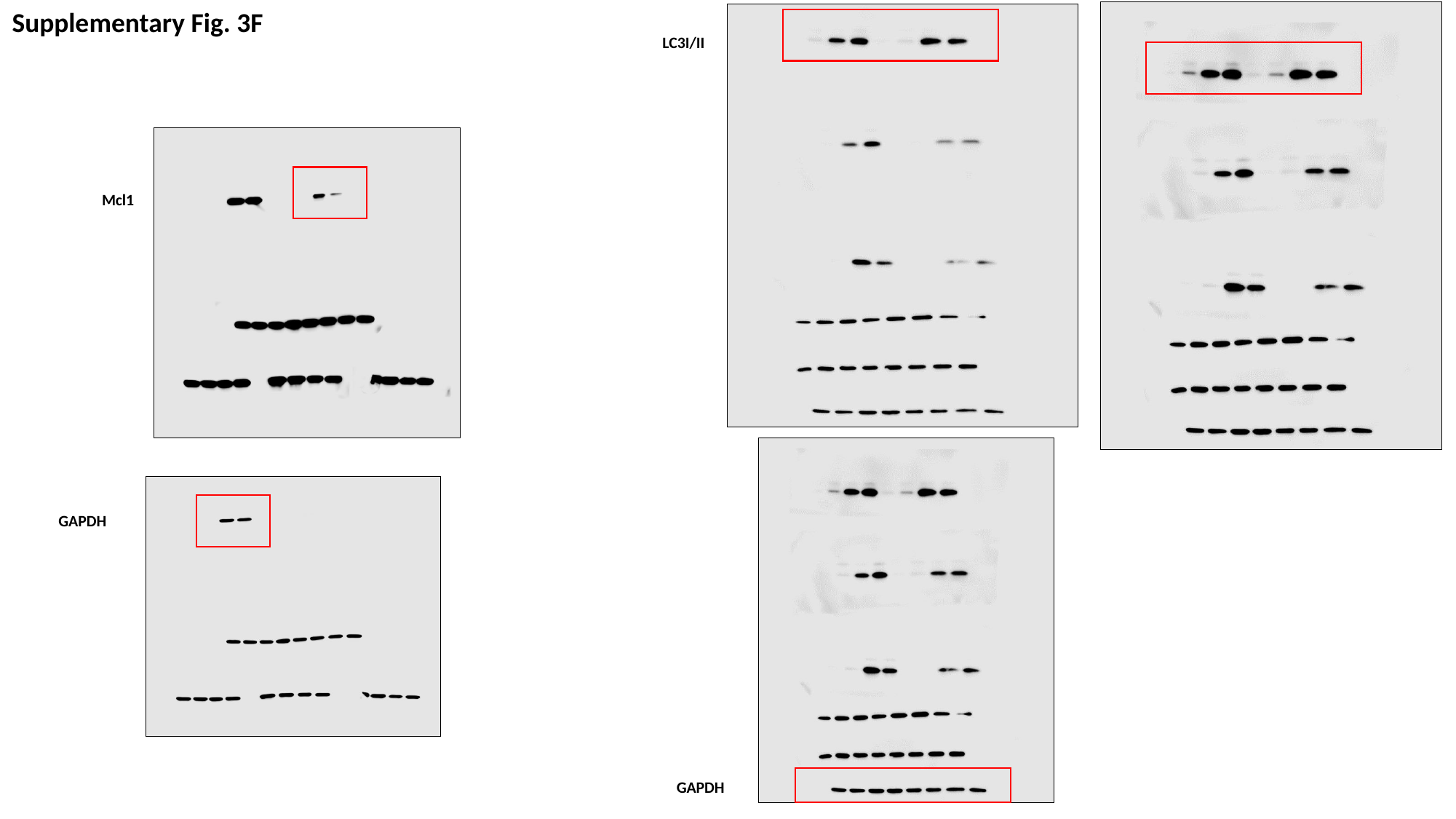

Supplementary Fig. 3F
LC3I/II
Mcl1
GAPDH
GAPDH

## Slide 37
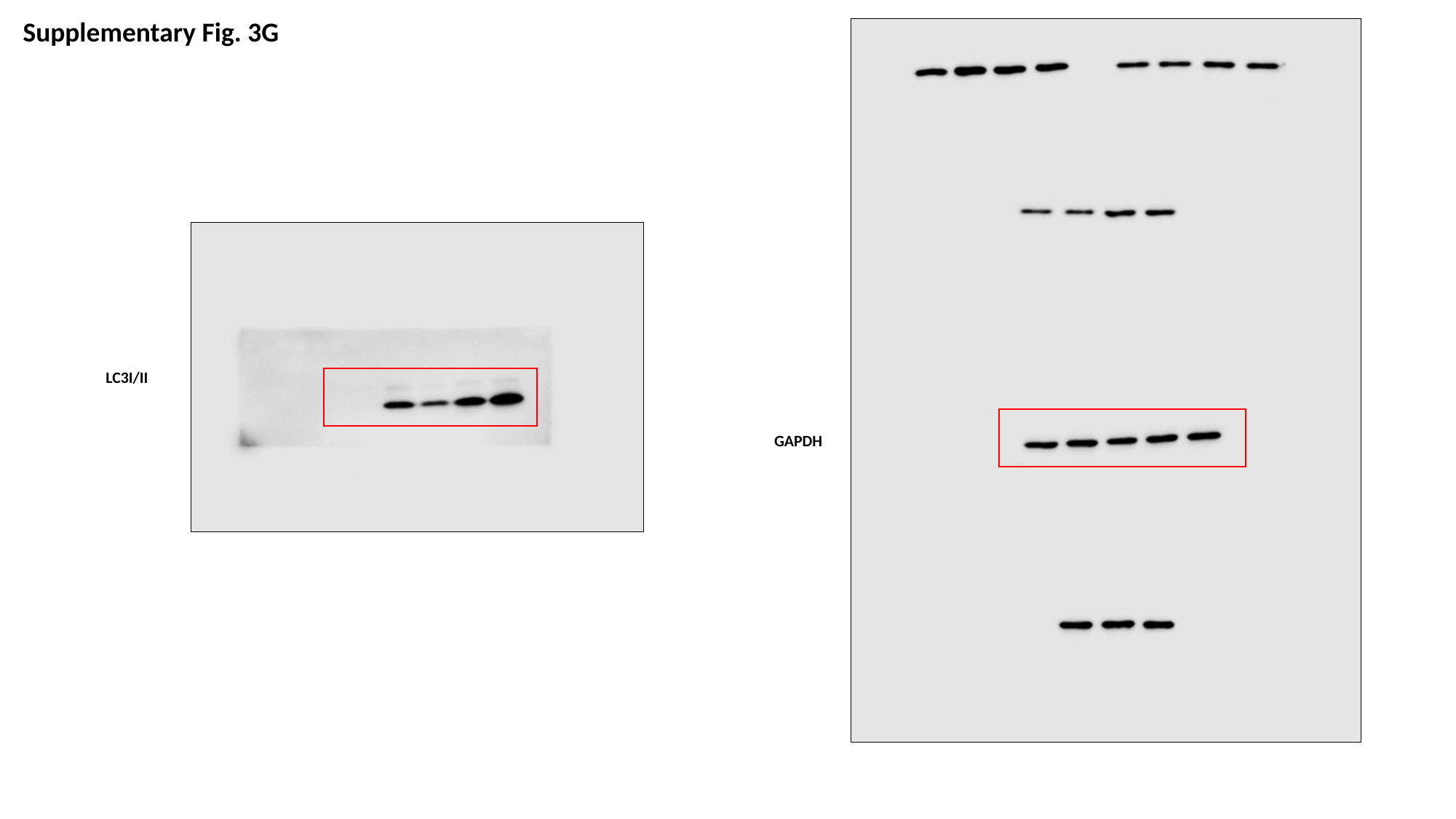

Supplementary Fig. 3G
LC3I/II
GAPDH

## Slide 38
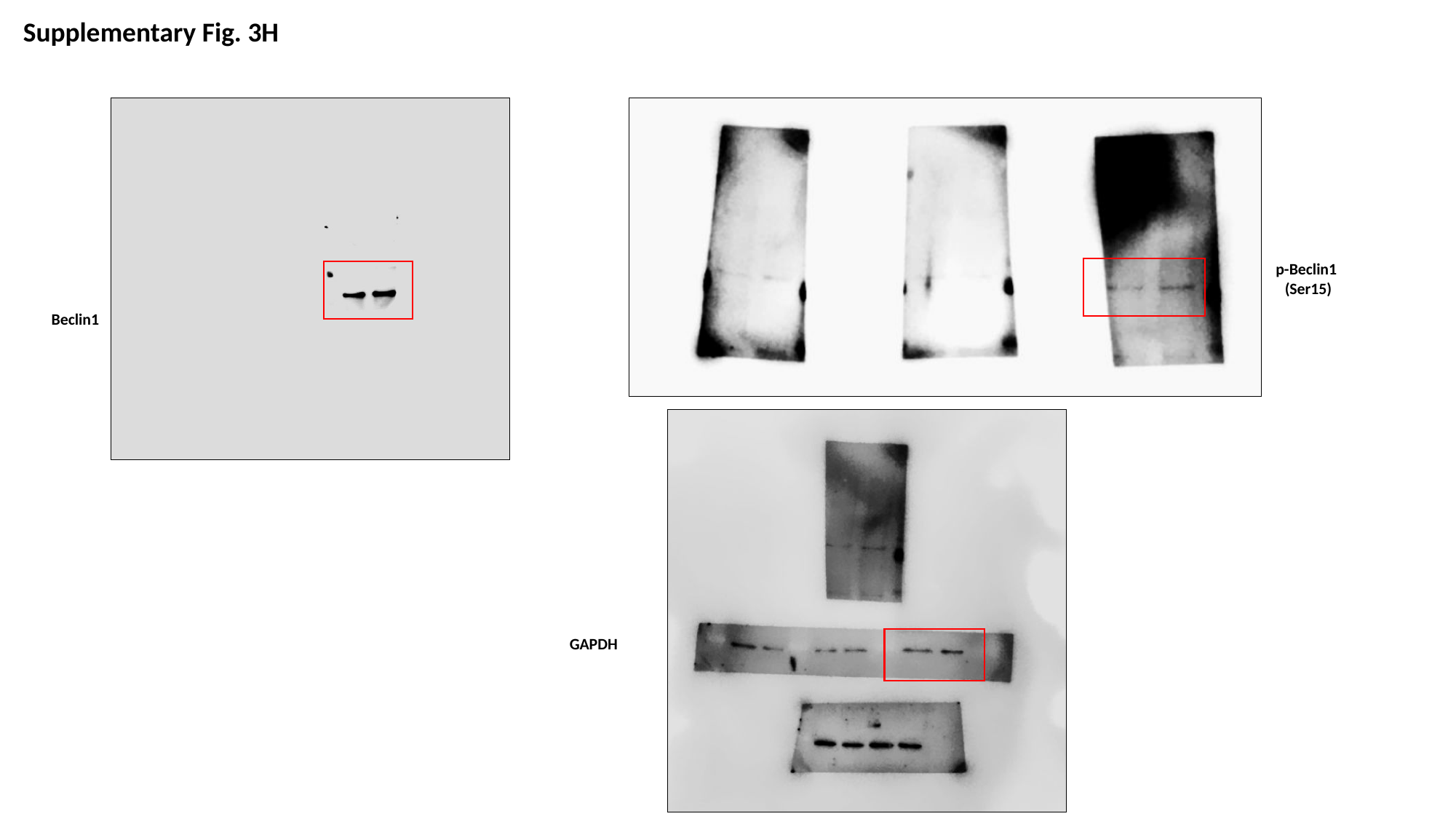

Supplementary Fig. 3H
p-Beclin1
(Ser15)
Beclin1
GAPDH

## Slide 39
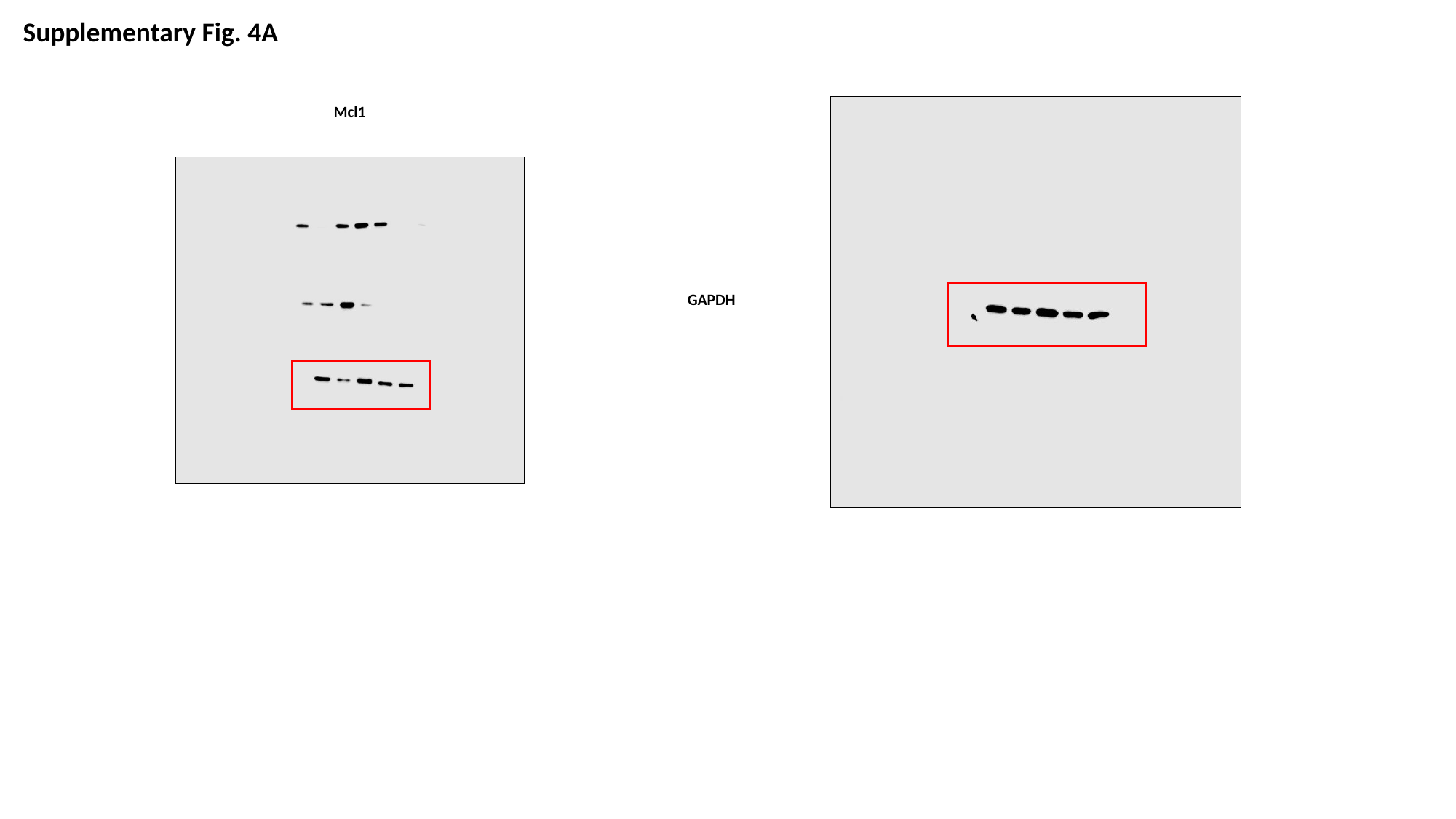

Supplementary Fig. 4A
Mcl1
GAPDH

## Slide 40
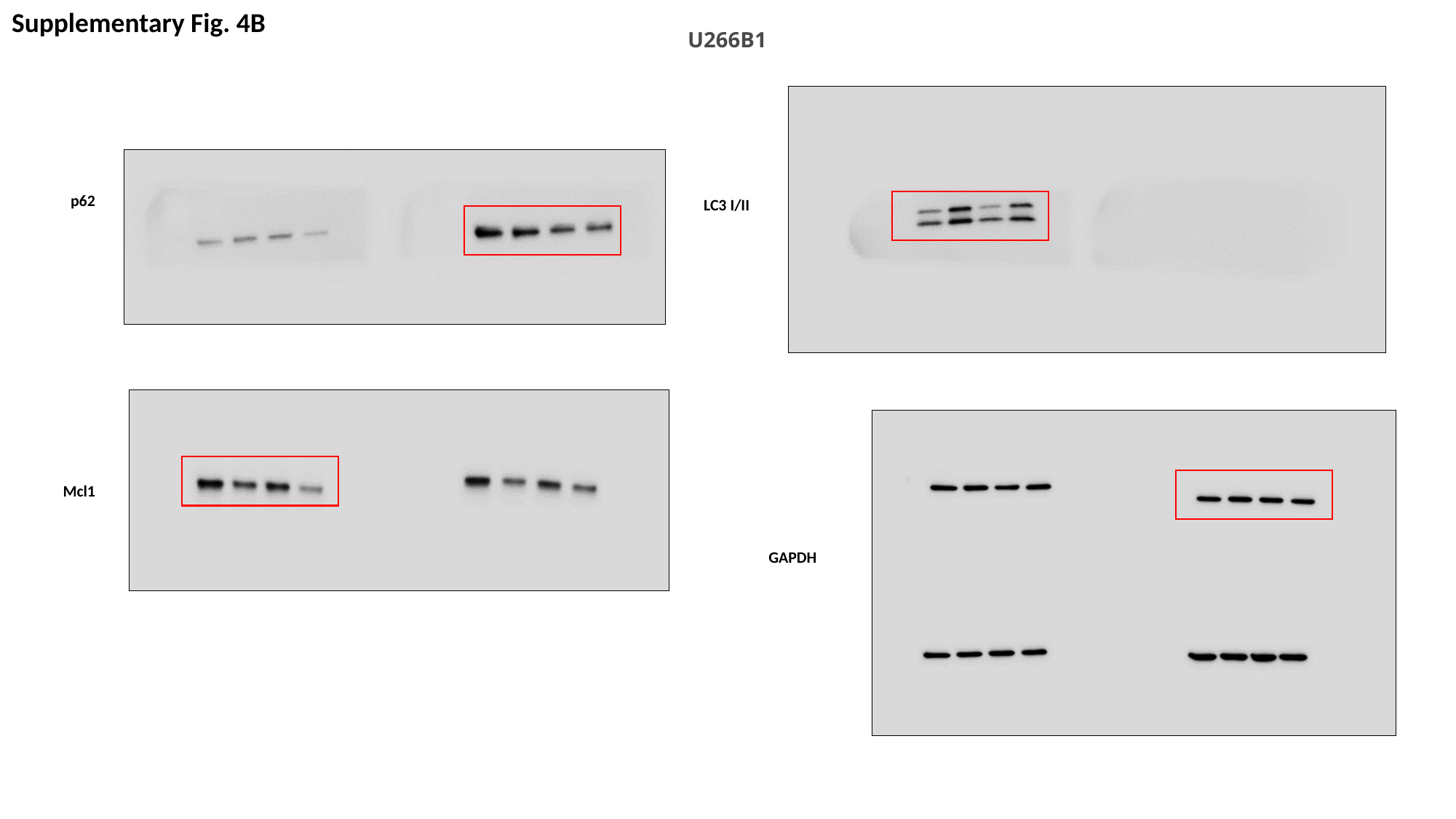

Supplementary Fig. 4B
U266B1
p62
LC3 I/II
Mcl1
GAPDH

## Slide 41
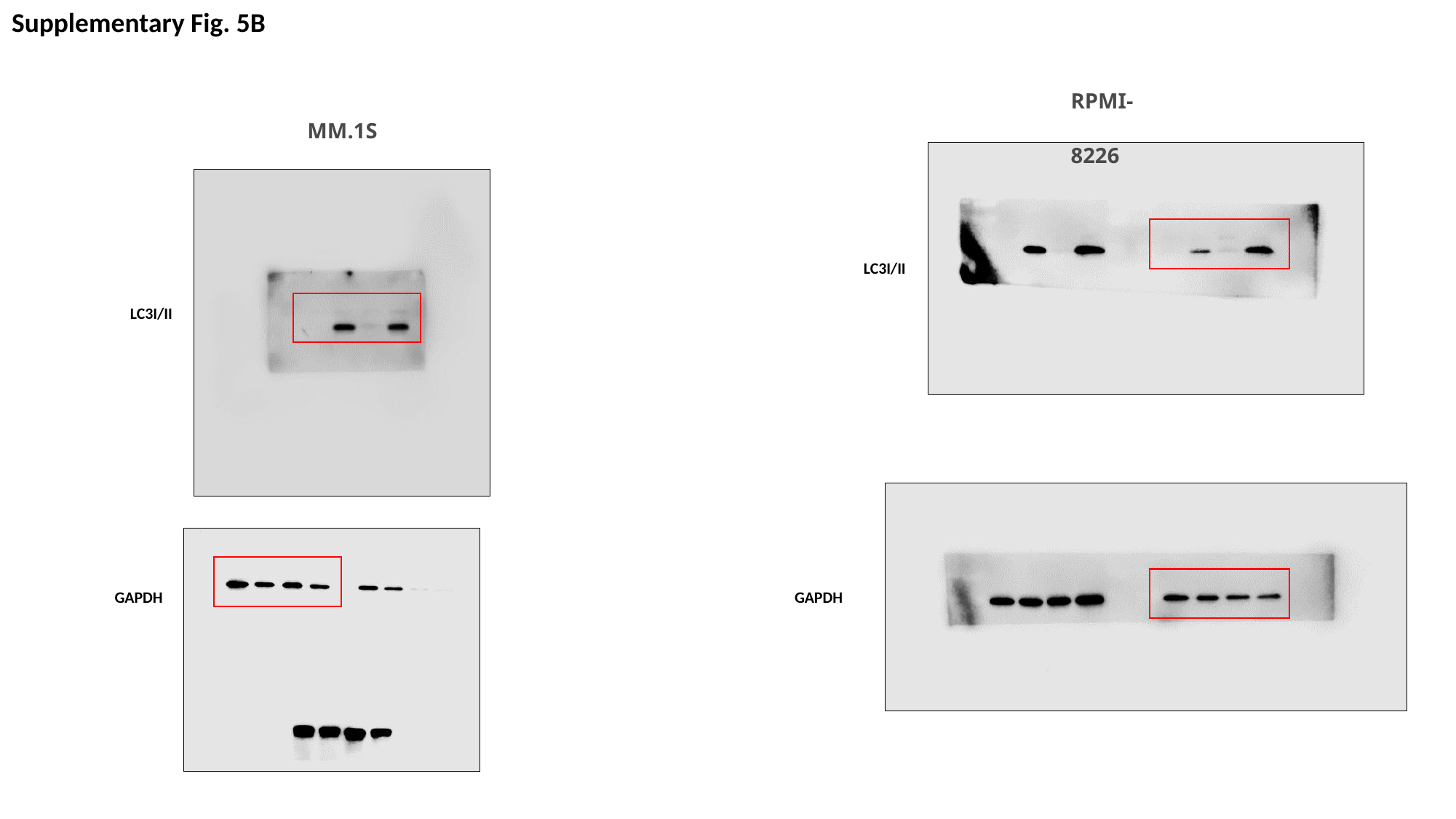

Supplementary Fig. 5B
RPMI-8226
MM.1S
LC3I/II
LC3I/II
GAPDH
GAPDH

## Slide 42
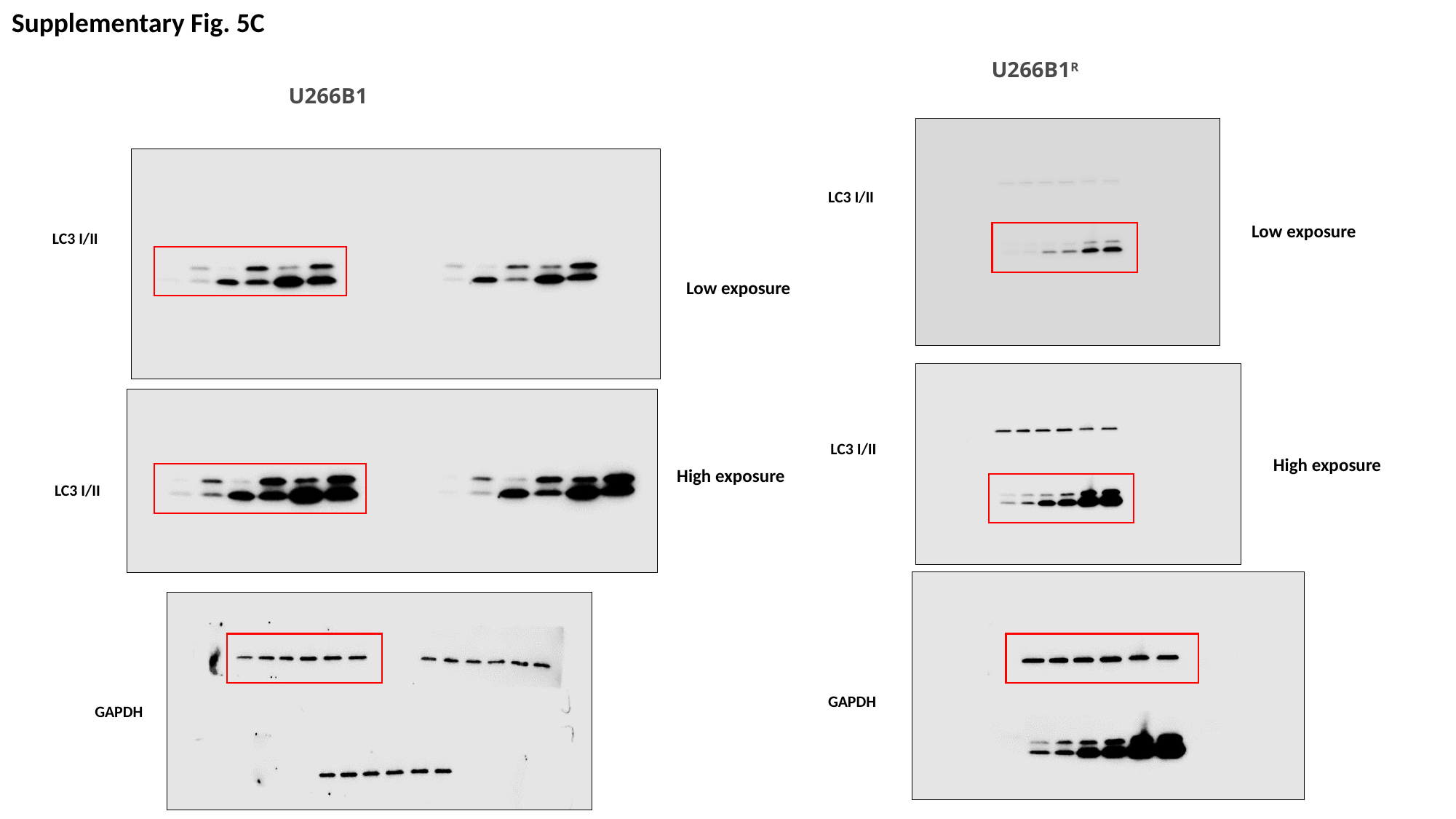

Supplementary Fig. 5C
U266B1R
U266B1
LC3 I/II
Low exposure
LC3 I/II
Low exposure
LC3 I/II
High exposure
High exposure
LC3 I/II
GAPDH
GAPDH

## Slide 43
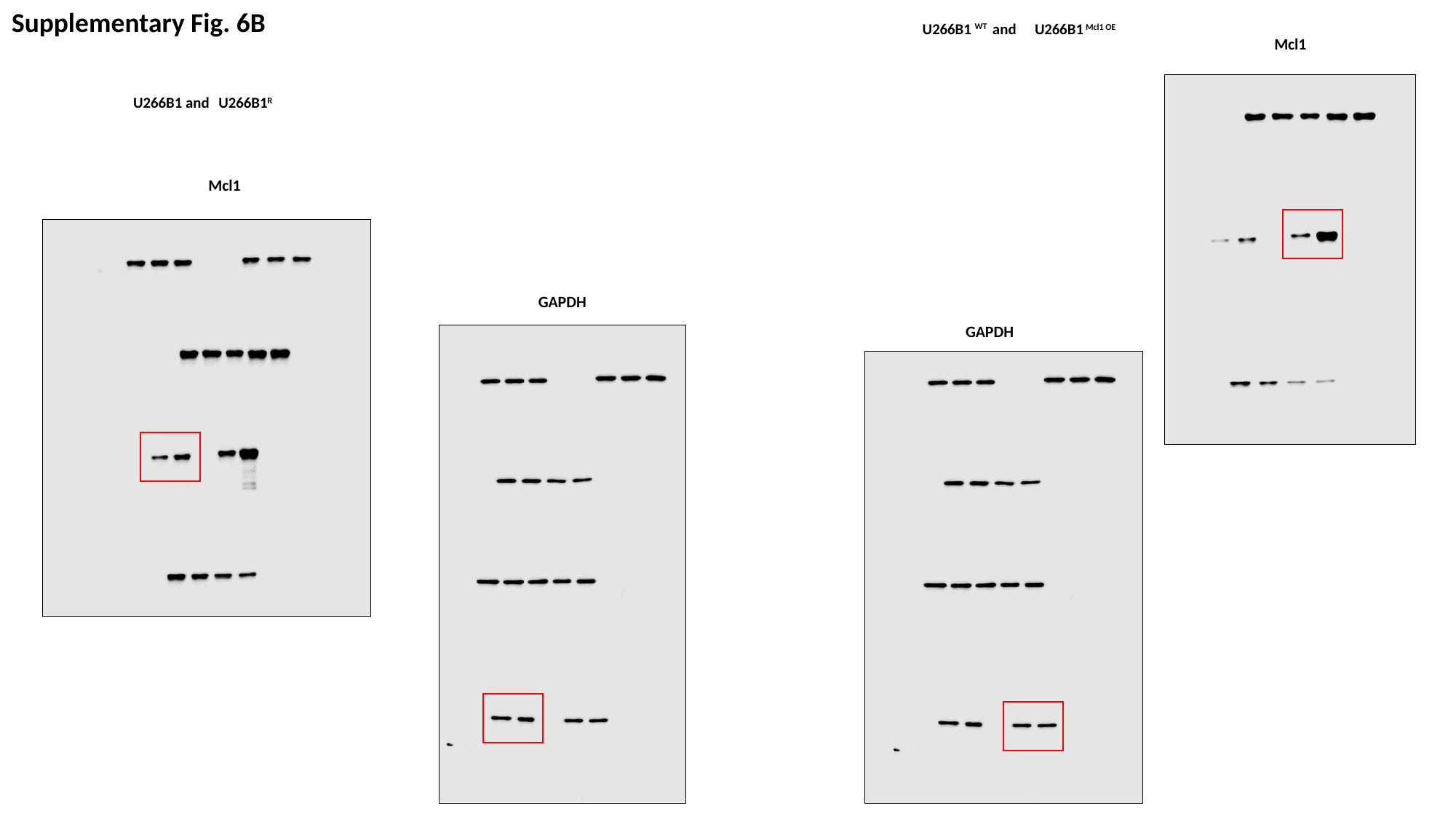

Supplementary Fig. 6B
U266B1 WT and
U266B1 Mcl1 OE
Mcl1
U266B1R
U266B1 and
Mcl1
GAPDH
GAPDH

## Slide 44
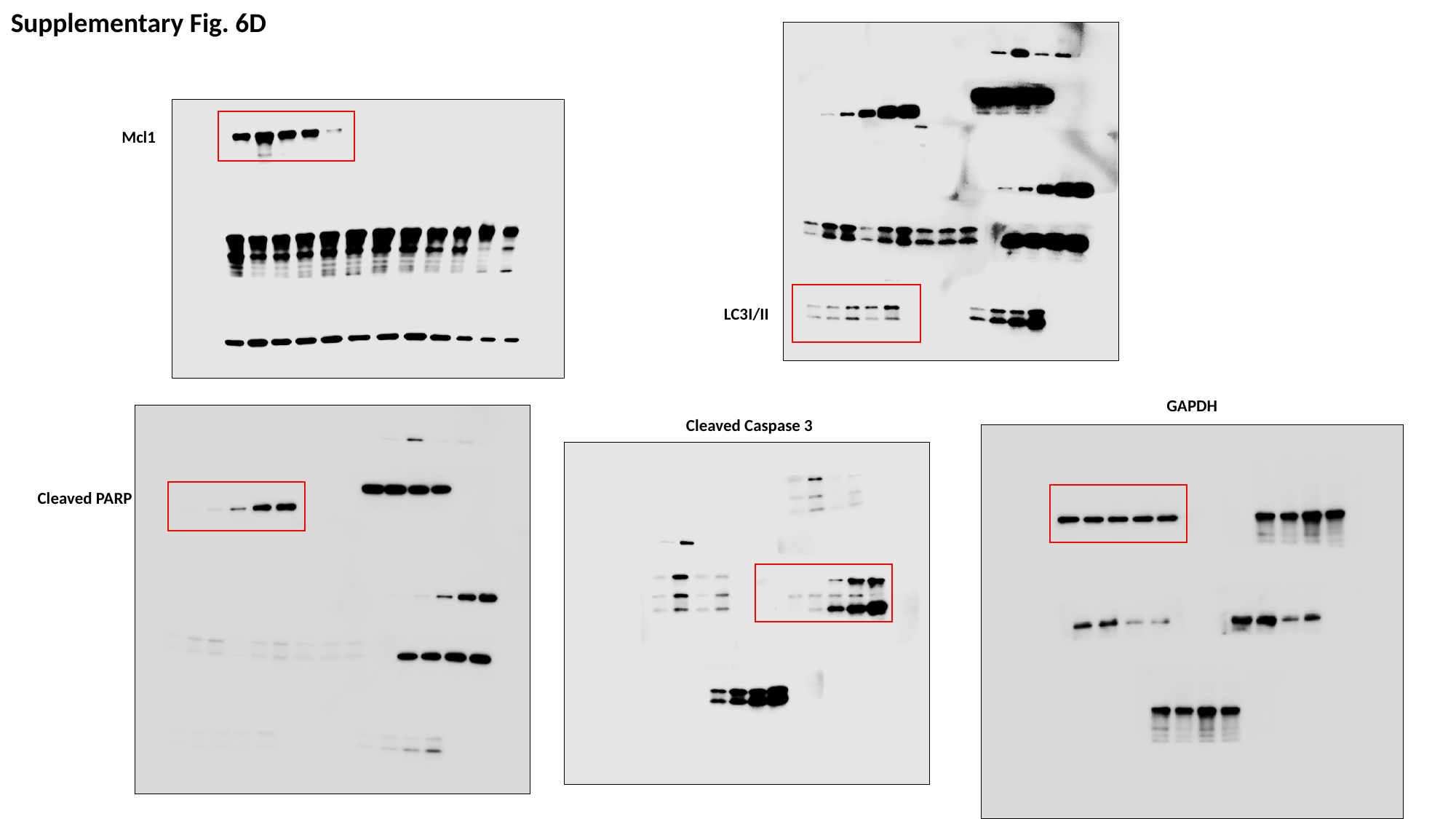

Supplementary Fig. 6D
Mcl1
LC3I/II
GAPDH
Cleaved Caspase 3
Cleaved PARP
